# Supplementary material for: ‘Unpacking’ pathways to lymphoma and myeloma diagnosis: Do experiences align with the Model of Pathways to Treatment? Findings from a UK qualitative study with patients and relatives
Source: BMJ Open. 2020 Feb 12;10(2):e034244. doi: 10.1136/bmjopen-2019-034244 (PMC7045052; doi:10.1136/bmjopen-2019-034244)
Supplement: Supplementary data [file bmjopen-2019-034244supp001.pdf]

Supplementary Table S1: Patient identification, diagnosis, sex, age, time to diagnosis (estimated) and relative's involvement

Patient ID	Diagnosis	Gender	Age[1] (Years)	Time to diagnosis (months)			Relative took part in interview (Y/N)
				T1[2]	T2[3]	T3[4]	
L01[5]	Follicular lymphoma	M	66	1	1.5	2.5	Y
L02	Diffuse large B-cell lymphoma	F	48	1	2	3	N
L03	Follicular lymphoma	F	63	1	3	4	N
L04	Diffuse large B-cell lymphoma	F	64	1.5	1.5	3	N
L05	Marginal zone lymphoma	F	57	0.5	3	3.5	N
L06	Follicular lymphoma	M	39	2	6.5	8.5	N
L07	Classical Hodgkin lymphoma	F	36	0.5	12	12.5	N
L08	Diffuse large B-cell lymphoma	M	60	13	2	15	Y
L09	Diffuse large B-cell lymphoma	F	62	0.5	6	6.5	N
L10	Follicular lymphoma	M	80	0.5	10	10.5	Y
L11	Mantle cell lymphoma	M	71	0.5	10	10.5	Y
L12	Follicular lymphoma	F	84	0.5	1.5	2	R
L13	Follicular lymphoma	M	63	12	3	15	N
L14	Classical Hodgkin lymphoma	M	33	0.5	4	4.5	Y
L15	Mantle cell lymphoma	F	75	6	2	8	Y
L16	Marginal zone lymphoma	M	64	9	5	14	N
L17	Classical Hodgkin lymphoma	F	37	12	1	13	N
L18	Follicular lymphoma	M	53	1	12	13	N
L19	Classical Hodgkin lymphoma	M	56	2	5	7	Y
L20	Diffuse large B-cell lymphoma	M	64	1	5	6	Y
L21	Diffuse large B-cell lymphoma	F	81	0.5	6	6.5	N
L22	Follicular lymphoma	F	52	0.5	2	2.5	N
L23	Classical Hodgkin lymphoma	M	23	0.5	3	3.5	N
L24	Diffuse large B-cell lymphoma	M	75	1	10	11	Y
L25	Marginal zone lymphoma	F	76	0.5	20	20.5	N
L26	Follicular lymphoma	F	63	8	6	14	N
L27	Diffuse large B-cell lymphoma	M	64	1	1	2	Y
L28	Marginal zone lymphoma	F	76	11	3	14	Y
L29	Diffuse large B-cell lymphoma	M	60	0.5	1.5	2	Y
L30	Marginal zone lymphoma	M	60	0.5	25	25.5	N
L31	Diffuse large B-cell lymphoma	M	65	2	15	17	N
L32	Diffuse large B-cell lymphoma	M	64	0.5	3	3.5	Y
L33	Marginal zone lymphoma	F	60	1	14	15	Y
L34	Mantle cell lymphoma	F	70	0.5	2	2.5	N
L35	Diffuse large B-cell lymphoma	F	69	8	1	9	Y
Myeloma 01[6]	Myeloma	M	67	1	2.5	3.5	Y
M02	Myeloma	M	63	2.5	7	9.5	Y
M03	Myeloma	M	71	1	17	18	Y
M04	Myeloma	F	78	1	5	6	N
M05	Myeloma	F	55	1	4	5	N
M06	Myeloma	F	71	3	1.5	2.5	Y
M07	Myeloma	F	56	1	8	11	N
M08	Myeloma	M	68	1	3	4	Y
M09	Myeloma	M	70	1	2	3	N
M10	Myeloma	M	74	2	0.5	2.5	Y
M11	Myeloma	M	59	3	15	18	N
M12	Myeloma	M	43	3	8	11	Y
M13	Myeloma	M	71	1	7	8	N
M14	Myeloma	F	59	1	8	9	Y
M15	Myeloma	M	58	1	17	18	Y
M16	Myeloma	M	66	3	15	18	N
M17	Myeloma	M	70	0.5	2	2.5	N

**M18**	Myeloma	M	71	1	11	12	Y
**M19**	Myeloma	M	62	1	1	2	Y
**M20**	Myeloma	F	74	7	5	12	Y

[1]Age at interview; [2]T1: Initial symptom/health change (estimated from information in the routine self-reported HMRN questionnaire) to first help seeking (appraisal and help-seeking interval); [3]T2: First help-seeking to diagnosis (diagnostic interval): [4]T3: Initial symptom/health change to diagnosis (total interval); [5]Lymphoma patient ID 01; [6]Myeloma patient ID 01.
