# Supplementary material for: ‘Unpacking’ pathways to lymphoma and myeloma diagnosis: Do experiences align with the Model of Pathways to Treatment? Findings from a UK qualitative study with patients and relatives
Source: BMJ Open. 2020 Feb 12;10(2):e034244. doi: 10.1136/bmjopen-2019-034244 (PMC7045052; doi:10.1136/bmjopen-2019-034244)
Supplement: Author's manuscript [file bmjopen-2019-034244.draft_revisions.pdf]

BMJ Open

BMJ Open is committed to open peer review. As part of this commitment we make the peer review history of every article we publish publicly available.

When an article is published we post the peer reviewers' comments and the authors' responses online. We also post the versions of the paper that were used during peer review. These are the versions that the peer review comments apply to.

The versions of the paper that follow are the versions that were submitted during the peer review process. They are not the versions of record or the final published versions. They should not be cited or distributed as the published version of this manuscript.

BMJ Open is an open access journal and the full, final, typeset and author-corrected version of record of the manuscript is available on our site with no access controls, subscription charges or pay-per-view fees (http://bmjopen.bmj.com).

If you have any questions on BMJ Open's open peer review process please email info.bmjopen@bmj.com

BMJ Open

‘Unpacking’ pathways to lymphoma and myeloma diagnosis: Do experiences align with the Model of Pathways to Treatment? Findings from a UK qualitative study with patients and relatives

Journal:	*BMJ Open*
Manuscript ID	bmjopen-2019-034244
Article Type:	Original research
Date Submitted by the Author:	11-Sep-2019
Complete List of Authors:	Howell, Debra; University of York, Health Sciences<br>Hart, Ruth; University of Edinburgh, Usher Institute of Population Health Sciences and Informatics; University of York, Health Sciences<br>Smith, Alexandra; University of York, Health Sciences<br>Macleod, Una; Hull York Medical School<br>Patmore, Russell; Castle Hill Hospital, Queen's Centre for Oncology and Haematology<br>Roman, Eve; University of York, Health Sciences
Keywords:	Myeloma < HAEMATOLOGY, Lymphoma < HAEMATOLOGY, Diagnostic delay, Help-seeking, Patient pathways, QUALITATIVE RESEARCH

SCHOLARONE™
Manuscripts

I, the Submitting Author has the right to grant and does grant on behalf of all authors of the Work (as defined in the below author licence), an exclusive licence and/or a non-exclusive licence for contributions from authors who are: i) UK Crown employees; ii) where BMJ has agreed a CC-BY licence shall apply, and/or iii) in accordance with the terms applicable for US Federal Government officers or employees acting as part of their official duties; on a worldwide, perpetual, irrevocable, royalty-free basis to BMJ Publishing Group Ltd ("BMJ") its licensees and where the relevant Journal is co-owned by BMJ to the co-owners of the Journal, to publish the Work in this journal and any other BMJ products and to exploit all rights, as set out in our licence.

The Submitting Author accepts and understands that any supply made under these terms is made by BMJ to the Submitting Author unless you are acting as an employee on behalf of your employer or a postgraduate student of an affiliated institution which is paying any applicable article publishing charge ("APC") for Open Access articles. Where the Submitting Author wishes to make the Work available on an Open Access basis (and intends to pay the relevant APC), the terms of reuse of such Open Access shall be governed by a Creative Commons licence – details of these licences and which Creative Commons licence will apply to this Work are set out in our licence referred to above.

Other than as permitted in any relevant BMJ Author's Self Archiving Policies, I confirm this Work has not been accepted for publication elsewhere, is not being considered for publication elsewhere and does not duplicate material already published. I confirm all authors consent to publication of this Work and authorise the granting of this licence.

For peer review only

'Unpacking' pathways to lymphoma and myeloma diagnosis: Do experiences align with the Model of Pathways to Treatment? Findings from a UK qualitative study with patients and relatives

Authors: DA Howell, RI Hart, AG Smith, U Macleod, R Patmore, E Roman

Abstract

Objectives: To explore experiences before lymphoma and myeloma diagnosis and whether these align with the Model of Pathways to Treatment[1] (MPT: incorporating symptom appraisal and help-seeking).

Design: A qualitative study with in-depth semi-structured interviews with patients and relatives. Interviews were transcribed verbatim, anonymised and analysed using qualitative description.

Setting: A UK population-based cohort of patients with haematological malignancies.

Participants: Fifty-five patients (35 with lymphomas, 20 with myeloma: diagnosed 2014-2016), and 27 relatives took part, within around a year of the patient's diagnosis. Participants were selected from those in the cohort who had returned a questionnaire about their symptoms and help-seeking, and consented to contact for further research. Sampling was purposive and designed to achieve maximum variation in age, sex and time-to-diagnosis.

Results: Participants described time from symptom-onset to diagnosis as ranging from several weeks to numerous years. Pathways largely aligned with MPT components and help-seeking could lead to the rapid identification and investigation of abnormalities. However, symptoms could be inadvertently interpreted as other conditions, which if perpetuated, could result in diagnostic delay. The latter was associated with chaotic pathways, with activities rarely occurring only once or in a linear sequence. Rather, intermittent or ongoing processes were described, moving forward through intervals (e.g. appraisal to help-seeking), and also backwards (e.g. help-seeking/re-presentation, back to appraisal). This is 'unpacked' within two themes: 1) 'appraisal and 2) 'help-seeking and re-presentation to diagnosis'. Various healthcare professionals were consulted, often on numerous occasions, as symptoms persisted, progressed or become increasingly alarming. Input from family and friends was substantial, as was the extent to which information seeking occurred.

Conclusion: Pathways to lymphoma and myeloma diagnosis align well with the MPT, although the repetition and complexity described is not entirely captured. Pathways were often difficulty and diagnoses delayed, despite the best efforts of patients, relatives and healthcare professionals.

For peer review only - http://bmjopen.bmj.com/site/about/guidelines.xhtml

Strengths and limitations of this study

- This is the first study to 'unpack' alignment of pathways to diagnosis of lymphoma and myeloma with the Model of Pathways to Treatment[1].
- This is the only study capturing the views of both patients and relatives, within contemporary healthcare systems.
- Findings are based on personal experiences and perceptions, described during in-depth interviews.
- Sampling aimed to achieve maximum variation and findings are likely to be largely transferable to settings with similar healthcare systems.
- The sample was derived from individuals returning a questionnaire about their symptoms and help-seeking, so the views of people unable to do this are not captured.

Keywords: Myeloma, lymphoma, diagnostic delay, help-seeking, patient pathways, qualitative research

For peer review only

For peer review only - http://bmjopen.bmj.com/site/about/guidelines.xhtml

1.0 Introduction

Early diagnosis of symptomatic cancer can lead to improved quality of life and survival[2,3], and has been a long-term UK policy priority, promoted via GP referral guidelines and waiting-time targets[4–6]. The impact of such measures has been variable, however, with substantial reductions in time-to-diagnosis seen for some cancers, but limited benefits for others; and some actually seeing increases[7]. Further targets and initiatives introduced in the recent NHS Long Term Plan[8] confirm this as an area that continues to be clinically relevant, with early diagnosis remaining one of the key areas in which UK cancer survival can be improved.

Arising in blood and lymph forming tissues, haematological malignancies (leukaemia, lymphoma and myeloma) are collectively the fourth most common cancer among men (after prostate, lung and bowel) and women (after breast, lung and bowel) in economically developed regions of the world[9–12]. With diverse treatment and outcomes, the World Health Organization currently recognizes over 100 heterogeneous haematological malignancy subtypes, which display a range of behaviours and outcomes[13]. Myeloma, for example generally presents in the bones and often requires intermittent treatment throughout a remitting-relapsing pathway more typical of chronic disease than cancer. Lymphomas (non-Hodgkin and Hodgkin, each having further subtypes), arise in various nodal/extra-nodal site(s) and may be indolent or aggressive. Myeloma and indolent lymphomas (e.g. follicular lymphoma) are usually monitored or treated intermittently with chemotherapy interspersed with observation; these diseases are considered incurable at diagnosis but can often be controlled long-term. Aggressive lymphomas (e.g. diffuse large B-cell lymphoma) are curable with chemotherapy[14,15].

Patterns of disease onset and time-to-diagnosis of lymphoma and myeloma are varied. For example, patients may be asymptomatic and diagnosed incidentally or they may have multiple and/or severe symptoms, that can be diverse and similar to other self-limiting conditions (e.g. fatigue, bone pain, swollen glands) to which they are often initially attributed; symptoms may also be insidious in onset or intermittent[16,17]. Importantly, time-to-diagnosis can be prolonged and associated with multiple GP consultations before hospital referral[18–22], myeloma being one of the cancers that has seen increases in time-to-diagnosis, despite policy initiatives[7]. Emergency presentation is comparatively common in these cancers, a route linked to advanced disease, more complications and poorer survival than other pathways[23–25]. Conversely, fewer two-week wait referrals are made to secondary care than for other malignancies, suggesting cancer is not suspected at referral.

Contemporary schematic models are now recommended for mapping and examining pathways to cancer diagnosis, along with checklists to ensure consistency in definitions, terminology, methods, data collection and reporting[1,26]. The Model of Pathways to Treatment (MPT: Figure 1)[1], for example, comprises a series of intervals beginning with appraisal (patient appraisal and self-management); followed by help-seeking (decision to consult a healthcare professional (HCP), and make and attend an appointment); then the diagnostic interval (HCP appraisal, investigation, and referral). Each interval is impacted by contributing factors, including patient actions, healthcare systems and disease characteristics. It is acknowledged, however, that MPT intervals may be non-linear, are not irreversible, and may require further 'unpacking' by cancer-type[1]. The current study aims to address this in the context of lymphoma and myeloma, by examining patient (and relatives') experiences in the time leading to diagnosis.

2.0 Methods

For peer review only - http://bmjopen.bmj.com/site/about/guidelines.xhtml

Qualitative methods were used as these are well suited to investigating how people make sense of their situation, and how and why they make particular decisions[27]. Details are outlined below in accordance with the consolidated criteria for reporting qualitative studies[28]. Our theoretical approach adheres to the principles of qualitative description[29,30], an applied method facilitating the production of concrete, low inference descriptions with practical relevance. The MPT[1] was used as a framework from which to examine activities within the appraisal, help-seeking and diagnostic intervals.

The study is based within the UK's Haematological Malignancy Research Network (HMRN: www.hmrn.org), detailed methods of which have been published elsewhere[31,32]. Briefly, HMRN is a specialist population-based cohort, initiated in 2004, that tracks entire treatment pathways on all patients newly diagnosed with haematological malignancies across 14 hospitals (catchment population ~4 million). Patient and Public Involvement is integral within HMRN, and takes place via a dedicated Partnership, which is overseen by a lay-committee and reports via a stand-alone website (www.yhhn.org). Patients routinely contribute to all HMRN funding applications, including the current study, in which they also participated in project design and take part in dissemination.

In addition to routine data collection, HMRN patients are asked to complete a questionnaire about their symptoms and help-seeking. Potential interviewees were identified from patients completing this questionnaire, who were aged ≥18 years, and within around 12 months of diagnosis. Sampling was purposive and designed to achieve maximum variation in age, sex and time-to-diagnosis. Ninety-two people were posted study material and invited to contact the research team if they wanted to participate; they were also invited to ask a relative/carer to contribute to the interview if they wished. Fifty-five patients (diagnosed 2014-2016; 20 with myeloma; 35 with lymphoma) and 27 relatives were interviewed (Table 1), with data collection continuing until saturation[33].

Interviews (conducted November 2015-May 2016, by RH & DH, who were previously unknown to participants), were semi-structured and guided by a schedule, developed from existing literature and clinical advice from within the study team (Table 2). With written consent, patients were asked to describe their pathways from first symptom(s) or health change(s) to diagnosis. Most patients were interviewed at home (two by telephone). Interviews lasted around 45 minutes and were audio-recorded, transcribed, anonymised and checked, with field-notes used to confirm accuracy and support data analysis.

Analysis was iterative, running alongside and informing data collection (RH, DH). After review and familiarisation with transcripts, narrative summaries were produced from the data and pathways mapped-out visually and shared with the research team (ER, AS). Several rounds of coding were undertaken, focusing on different aspects of the data, including activities and events occurring within intervals defined in the MPT. Constant comparison (codes with codes, codes with data, codes with theory) and memoing[34] supported refinement of codes and identification of patterns and relationships. Codes and emerging themes were discussed by the team and refined until consensus was reached.

3.0 Findings

Participants described time from symptom-onset to diagnosis as ranging from several weeks to numerous years. Within this disparate time-frame, pathways largely aligned with MPT intervals,

For peer review only - http://bmjopen.bmj.com/site/about/guidelines.xhtml

events, processes and contributing factors, and help-seeking could lead to the rapid identification and investigation of abnormalities. However, experiences did not always fit the distinct compartmentalisation depicted in the MPT. More chaotic pathways were described, often if symptoms were inadvertently interpreted as other conditions. In this case, activities rarely occurred once or in a linear sequence, but required intermittent or ongoing processes, whist moving forward through intervals (e.g. appraisal to help-seeking), and also backwards (e.g. help-seeking/re-presentation, back to appraisal). This was common This is 'unpacked' within two themes: 1) 'appraisal', in which appraisal, re-appraisal and self-management; and 2) 'help-seeking, re-presentation and diagnosis'. Input from HCPs, family and friends was generally said to be substantial, as is the extent to which independent information seeking occurs. Each theme is described below using illustrative verbatim quotes, and summarised in Figure 2.

3.1 Appraisal

3.1.1 Appraisal and reappraisal

Patient appraisal of symptoms and health changes was described as ongoing by participants, with observation and monitoring occurring periodically or continuously from first symptom to diagnosis. When 'normal' health was not regained, appraisal was said to become more deliberate and rigorous. It involved, for example, checking and comparing affected and non-affected sites: *'My mind was going, "If you can feel* (a lump)...*both sides, then it's normal. If you can feel it on one side, then that's something to worry about". So I tried, I felt the other side, and again…'* [Lymphoma patient 06: L06]; usually over time: *'I couldn't decide whether my skin was a different colour…I'd look at it and think, "It looks strange", and then I'd look again, and it didn't'* [L03].

Interviewees described re-appraising persistent, progressive or new symptoms, which could be perceived to be related, or unrelated, to the initial problem. Unremitting symptoms sometimes led to more precisely and formally appraisal, including documentation of health changes, pain, medication and weight: '*I kept a note, like week-by-week almost*'…[L25]. A key element of appraisal involved patients and relatives considering numerous explanations over time for symptoms: '*Could I have ME?'* [L05]; *'Maybe it was an ulcer'* [L26]; *'I was just thinking, "actually, is this becoming a mental health issue? Is it first signs of dementia?"'* [Relative of myeloma patient 20: M20].

Interviewees described information seeking from various sources during appraisal/re-appraisal, particularly for ongoing symptoms; an issue not wholly acknowledged in the MPT. One myeloma patient consulted relatives about their family history (osteoporosis) as a potential explanation for symptoms. Others described questioning medically experienced friends, colleagues or acquaintances: *'There's a doctor…lives further down the lane…I said* [to him] *"I don't really like asking but…"'* [L03]. Many explore symptoms via the internet: *'I'd looked at all sorts of problems I thought* (patient) *might be having with bones"'* [Relative, M20]; which was more effective when symptoms were typical of myeloma or lymphoma: *'I think I put in something like "itchy skin, night sweats, lump in neck"; Hodgkin's lymphoma came up, through a few different clicks'* [L14]. This could lead to rapid help-seeking/re-presentation: *'had I not seen* (information)*, I'm not convinced I would have said anything to the doctor, but because I'd seen it and I thought, "Crying out loud…", I was like, "We've got to do something…someone needs to put all these pieces of the puzzle together"'* [Wife, L14]. It could also lead to emergency presentation: *'the more I read* (about lump)*, the more I was*

For peer review only - http://bmjopen.bmj.com/site/about/guidelines.xhtml

scared, and I just went to A&E…'" [L23]; or denial: *'Well, when I started reading about cancer I just switched it off, because I thought, "No, no, that's not right"'* [L05].

Considerable involvement of relatives, friends and colleagues in appraisal/re-appraisal was described, though not wholly depicted in the MPT: *I just looked at his tummy and I thought, "that's an odd shape, that", and it just flashed through my mind and I thought "That could be a tumour"'* [Wife: L20]. Relatives sometimes said they adapted their own behaviour to accord with the patient, often over long time-periods: '(Husband's) *height loss, was gradual, at first, so, I noticed…I stopped buying shoes with heels, because I was getting to be, you know, too tall* (for him) *when I was wearing shoes with heels…That had probably been going on…maybe a couple of years'* [Wife, M18]; or they began to discuss the patient's symptoms and health changes between themselves: *'We knew there was something wrong…my daughter actually said, "You know, dad's not right at all, is he?"'* [Wife, M02].

3.1.2 Self-management

Self-management was not solely associated with the appraisal interval, but was described as occurring throughout the pathway, particularly if patients or HCPs had attributed initial symptoms to self-limiting or life-course issues (e.g. age, menopause): *'we're the kind of people who go to the doctor as a last resort, and we'd tried the obvious things ourselves, you know* (lists remedies for indigestion)*, anything you think might ease it. But it didn't…'* [Wife, L20].

Some patients said they used distraction techniques for relief, often long-term: '*I wake up because my tummy's hurting, and then I get up and start the day, and then I try and fill my day with things, so that I don't think about it…and, that was how it went and, we're probably talking a couple of years of that…just putting up with these different things'* [L26]. Others reduced or stopped activities viewed to cause/aggravate problems: '*You'd stopped driving for about two months (before diagnosis), because you were in…pain'* [Wife, M03]; [Interviewer: 'You gave up work 18 months ago…?'] '*Yeah…it were getting a little bit difficult for me, and I didn't want to embarrass myself'* [L16].

People described using aids over long time-periods: '*I used to go to work with that belt* (meant for weight-lifting)*, you know, on my back'* [M15]. Others used sticks, wheelchairs or riser-chairs to remain mobile. Lifestyle changes were made: *'I've had grumbling problems… probably at least two years…the kind…you treat yourself, with indigestion medicine, drinking water, trying to eat well, going for exercise, all those things that you think "This'll sort me out. I'm just getting a bit older and I need to look after myself"* [L26]. Some took supplements *'we were having Complan drinks with whole milk…protein drinks, powders, anything to get something into his system…the weight back'* [Wife, L20]; or self-medicated with analgesics, anti-inflammatories, anti-histamines and gargles.

Long-term strategies were reported to manage debilitating tiredness, including napping or sleeping more: '*I were feeling tired all the time…I used to eat my sandwiches before my lunch…as I went round, and then as I stopped the van, I used to turn the ignition off, and lay on the seat, and go to sleep'* [L16]; *'I tended to sleep a lot. I'd come home for lunch at 12 o'clock…fall asleep in the chair… almost every day…'* [M12]. *And then you'd come home and fall asleep, and you'd wake up and have tea, then fall asleep again, and then wake up for a couple of hours watching telly, and then go to bed*' [Wife, M12].

3.2 Help-seeking and re-presentation to diagnosis

For peer review only - http://bmjopen.bmj.com/site/about/guidelines.xhtml

Concurring with the MPT, all patients described help-seeking for symptoms. Some said this led to the immediate identification of abnormality/suspected cancer, which was rapidly investigated and diagnosed: '*The doctor…was lovely and she examined* (patient)*…and said "I can feel something, in there, and she booked him in…for a CT scan…endoscopy, yeah, and a blood test…on the scan they saw, there was a mass'* [Wife, L20]; '(GP) *did a very thorough check…he's always been very through...physical and, yeah, mental if you like…I've always found him absolutely superb.* (He) *listens not just to what you say, but what you don't say and, and sort of, reads the signals;* [Relative]: (GP) *decided it was something serious, and then you were sent for a scan…you had a biopsy, and it all happened very quickly*' [L01 & Wife].

Alternatively, other conditions were initially suspected, particularly if these had symptoms in common with the cancer: *'I noticed a swelling at the top of my groin…*(GP) *said "that feels like a, a hernia to me…I'll sort you out an appointment to get you referred"'* [L06]. Premature diagnoses such as these could be quickly re-categorised, or perpetuated and delay re-presentation: *'I noticed, like, a little pea-sized lump in my leg…my right calf…I thought…"Probably me varicose veins". I didn't rush, I went to see the doctor, regarding something else, and I just happened to mention to him about it… he had a feel of it, and he says "right, we'll send you off to have and ultrasound", you know, get it checked out. I asked* (radiographer)*… "Is it a varicose vein?" She says "yes". 'I got on with my life, I just carried on. I thought well fair enough, you know, its varicose veins…I never bothered when I started getting other lumps in my leg, I thought, "well it's all just part of it".* (The following year) *this lump was steadily growing…I started getting other lumps…the lumps joined together…starting to turn red and poke out of my skin...It just sort of exploded sort of thing, you know, it was frightening how fast it was growing…I went to see the doctor again'* [L31].

Initial help-seeking could include requests for broad health-checks*: 'I just felt, okay I'll go to the doctor's and…*(say)*, "Well, I want an MOT…I seem a bit tired"…and it was a locum, and I explained my symptoms, and he sort of said, "Well, they seem a bit of an odd set of symptoms, so we'll give you…full blood tests", and thank goodness he did'* [M05]; *'I did sort of ask for like a 'well woman' check, because I didn't in my mind feel like I was right'* [L17]. General interventions were also sought: *'I was just really feeling fed up, because I was so tired…I went for a tonic, I said, "Can you give me just something to pick me up?"'* [L34]. Some patients said they gave HCPs only partial information about their symptoms, either purposely (only reporting symptoms they thought the HCP would considered important) or inadvertently: *'I didn't mention my general apathy, because I just thought it was just apathy to be honest'* [M12]*; 'many's the time I've come out thinking, "Oh damn, I didn't mention that"' [L24]*. Interviewees said this could have delayed their diagnosis: *'maybe I wasn't forthcoming with the information'* [M03].

Re-presentation was extremely common and occurred quickly with increasingly alarming symptoms: *'(I) went to the bank, and wet myself… and I thought, "Oh my God… what's going on here?" And I went to the GP'* [L09]; *'the sickness…he started bringing his evening meal up, and unable to sleep at night, the pressure on his tummy…he'd be walking the floor, you know, just generally, obviously something very wrong* [L20].' It could be delayed if the patient was told resolution may take time: *I saw two* (GPs)… [Interviewer: Who said?] *'Give it time'* [M13]; or if symptoms were intermittent: *'he'd be ok for a while, and then, you know, maybe a few nights he'd be sick'* [Wife, L20]; *'I carried on working, as you do…I didn't feel too bad really, some of the time'* [L20].

Re-presentation often occurred on multiple occasions: *'I went back probably about three times between February and August…* [M13]; '*all that time, just going backwards and forwards to the doctors*' [Wife, L24]. Sometimes this was associated with ongoing normal investigations: '*I just felt so*

For peer review only - http://bmjopen.bmj.com/site/about/guidelines.xhtml

tired. I'd just no energy, you know, and the daft part is, I think I'd three lots of blood tests done as well, to try and find out, you know, and yet none of 'em showed anything to do with the lymphoma' [L31]; '*The blood test revealed nothing, did it, and neither did the camera in the stomach*' [L20]. Where abnormal blood tests were noted, rapid hospital referral was described: '*I'd had about four or five different blood tests, and it were all coming back negative'* [M15]…'*til these blood tests did come back abnormal, all of a sudden, so that's when they sent him up to haematology* [Wife, M15]; '(GP) *immediately, more or less said, I'm sending you into hospital. You're anaemic*' [L24].

Re-presentation sometimes occurred at the request of HCPs: '(GP) *said to me if there are ever any changes with the lumps to inform him*' [M12]. This was sometimes associated with HCPs expressing frustration about their inability to explain symptoms: '(GP) *said: "come back in four weeks…I'm getting me books out…I don't understand what the symptoms are pointing me to*" [L24, paraphrasing GP]. Interestingly, this patient's main concern ('*night urination…combined with the loss of weight, that was what really got me, erm, worried'*), remained unresolved after successful treatment, and at this point was considered unrelated, with urology review awaited.

Some interviewees described requesting more specific input from their HCP at re-presentation, including direct appraisal: '*you need to see my neck again'* [L14]; '*have a look while I'm standing up*' [L34]; '*I said to (GP)…"Can you actually measure me?" which she did…she indicated… that I'd lost about two inches'* [M14]; and particular investigations: '*I said, "Look, there's something wrong here. I need an X-ray"'* [M13]; *'I said "I'm not picking up" and she agreed (to blood tests)'* [L05].

Re-presentation also occurred due to a growing conviction something was really wrong: *'Normally I'm not somebody who goes to* (the) *doctors…it was just a feeling that something definitely wasn't right. Despite getting the first opinion'* [L01]; *My mobility was shocking by then…I was having great difficulty in doing anything, literally and I knew then that there was something…*' [M03]; '*I weren't going to take "no" for an answer… because I knew there was something'* [L34]. Doubt about an earlier explanation was often key: '*I was convinced there was something wrong with me, other than osteoporosis, because I thought "I shouldn't feel, this unwell, with osteoporosis."'* [M14].

Evidence (e.g. diaries) was often produced by patients at re-presentation to demonstrate the extent and severity of symptoms: *I thought, "*(GP's) *probably thinking I'm just a hypochondriac". But when I got the evidence of the weight that I was losing, I didn't feel that…*(GP) *could turn a blind eye to that.'* [L25]. New information from re-appraisal (symptoms, mismatch between symptoms and preliminary diagnoses, and test results) or internet searching was also said to be passed on to HCPs: *'I said, "I think I've got lymphoma…I seem to have these lumps that haven't gone down at all…and I think now I'm getting, it's either anxiety, or I'm now getting B-symptoms, which is night sweats…"'* [L06]. This could prompt action: *'I went back to the GP and said, "Well,* (ENT) *say it's TB or lymphoma", and the GP said, "Well, I don't think it's lymphoma, but, if they're saying TB, I'll send you for an X-ray"'* [L07]; but not always: *'I'd been in pain for a long time, and I actually said, "I think I've got cancer" and (Dr) said to me, "You don't look like* somebody *who's got cancer"'* [M14]; '(GP) *said, "No, no, you haven't…lymphoma, old people get lymphoma. You're (under 40)…don't even think about that"'* [L06]. Patients reported chasing-up information to give to HCPs (e.g. results): *'I rang* (surgery)*, three times a week, every week, to see if they'd had a reply*' [Wife, L11].

Lack of progress towards diagnosis could lead to distress, anxiety and frustration at re-presentation: *''I was getting towards my wits' end, because I knew that there was something wrong, and I just felt as if* (HCPs) *weren't taking me seriously*' [L06]. Occasionally, this resulted in emotional outbursts:

For peer review only - http://bmjopen.bmj.com/site/about/guidelines.xhtml

'The doctor said, "Have some antibiotics"… and we broke down and were just like, "This isn't right". So he called the hospital and got (Husband) *into hospital'* [Wife, L14]; *'They thought it was anxiety, because I'd got myself in a state, and I was in a tearful mess at the doctor's, because of feeling ill and they're not getting anywhere…So she just thought it was an anxiety issue. It was like turned from one thing to something else'* [L17].

Re-presenting as an emergency (e.g. via A&E) was not uncommon, often for worsening symptoms and to gain rapid healthcare. This could be instigated by HCPs, the NHS '111' helpline, or patients and relatives: *'I was getting so desperate…I knew something was wrong, and I felt that nobody was really listening to what I was saying. I did actually go to A&E a couple of times. Well, once under our own steam, and once we rang 111, and they said "Go to A&E"'* [M14]; *'I saw the nurse, and she said, "Look, you know, go to A&E and let them have a look at it, so that's what we did'*. Symptom complexity could result in consultation with multiple specialists even via this route. One patient, for example, was informed "it's a tumour" after assessment in A&E by a physiotherapist, vascular surgeon and plastic surgeon [L31]. Some said they asked HCPs for hospital referral/admission, which was occasionally disputed: *'"Oh no, she doesn't need to go to hospital"'* [Husband paraphrasing HCP, L35]; or a GP-instigated referral was rejected by hospital administration.

A few people described feeling obliged to '*stick with*' the first GP consulted: '*he knew the situation, and I kind of felt like, it'd have been going behind his back to go and see another GP'* [L07]. Others did not: *'The very first doctor I saw. I saw him twice and then I knew after that I wasn't going to ask for that particular doctor'* [M14]. Some reported re-presenting to HCPs they knew and trusted, were more experience, or just different: *'We would sometimes just ask for a different one, just to get a different opinion'* [L34]. Various other specialists were consulted: '*I'd started to suffer with mouth ulcers, and…I actually went to my dentist'* [L30] and advice was sought at unrelated hospital appointments: *'I went to see my Crohn's surgeon… my normal check-up, and I mentioned (the lump) to him, and he's the one really who pushed it forward fast…'* [L32]. Complementary therapists were accessed, with mixed results: *'They did send me to physio…*[M15]*… eventually, he were in that much pain, he couldn't go'* [Wife, M15]; '*I went to an osteopath…which did help'* [M12]; '(physiotherapy) *made matters worse'* [M18]. An '*herbalist' and an 'allergist'* were consulted; and one person considered '*colonic irrigation*'.

Relatives and friends were said to influence help-seeking and re-presentation. This could be encouragement: '*My mum and friends* (were) *saying, "You…really ought to go to the doctors"'* [M05]; or discouragement: *'I had these lumps on my neck and I, I didn't take any notice of them…I said to a friend and she said…"Oh it's nothing, everybody gets lumps"'* [L15]. Relatives were said to attend GP appointments with patients, often to advocate on their behalf: *'you become increasingly dependent on somebody to go with you…for reassurance and support'* [M14]; *'We made and appointment…we had to insist* (patient) *needed to see someone, and we went together, and we said, "we need help here"'* [L20]; *'"I'm not waiting for this appointment", I said, "because (Husband, the patient) can't wait". I said, "I want him to see* (haematologist)*, as soon as!" I said, "I know you can do it, because I worked in the health-service" so she said, "Yes, okay". Otherwise, I honestly thought he could sit in the chair and just go* (die)*. He was so ill'* [Wife, L11]. Some people said they decided to re-present via private healthcare, paying for specific treatments (e.g. physiotherapy), investigations and/or hospital referral, and considering this key to diagnosis. Access to private healthcare was said to be rapid: '*it bought me time'* [L13]. Where GPs knew patients had private insurance, they were reported to suggest referral *'I went backwards and forwards to my GP, and I'm covered by my husband's private hospital thing, so* (GP) *suggested that I went to see a consultant back specialist'*

For peer review only - http://bmjopen.bmj.com/site/about/guidelines.xhtml

[L09]. More commonly, patients portrayed private care as an extreme but necessary measure: *'the timeframe for me was…it was too much, so I said "I want a private referral"'* [M12].

4.0 Discussion

Principal findings

Pathways to lymphoma and myeloma diagnosis aligned well with the MPT, with most patients experiencing key aspects of this model (i.e. symptom onset, appraisal, help-seeking, hospital referral and diagnosis)[1]. However, further 'unpacking' confirmed that, often despite the best efforts of patients, relatives and HCPs, pathways were often much more complex and chaotic than depicted in the model, with activities rarely being described as occurring on a single occasion or in a linear sequence. Rather, intermittent or ongoing processes were described, that moved forward through intervals, but also backwards. Repeat or continuous re-appraisal was said to occur along with multiple re-presentations to HCPs, which could cause anxiety and distress. Furthermore, self-management did not occur solely within the appraisal interval, but across pathways. Input from family and friends was substantial, as was the extent to which independent information seeking occurred. Events at one time-point (e.g. reassurance about symptoms) was said to influence later actions and time-scales (e.g. delayed re-appraisal/re-presentation). Similar experiences were reported by patients with myeloma and lymphoma, and various HCPs were consulted during the diagnostic interval.

Strengths and weaknesses

This is the only qualitative study exploring pathways to diagnosis of lymphoma and myeloma in the context of the MPT, whilst taking an overview that tries to capture the interplay between intervals, people and events. The sample is comparatively large (55 patients; 27 relatives) and in-depth discussions ensured findings were based on personal experiences and perceptions. Inclusion of relatives is a unique feature and a particular strength, as these individuals were able to contribute their own views of the pathway and describe their involvement in events. Interviews were conducted within a year of diagnosis and use of letters, calendars and diaries was encouraged to promote recall. Although generalisability is not an overarching aim of qualitative research, maximum sample variation increased the transferability[35] of findings to places with similar healthcare systems, though experiences may differ in countries without universal provision, or where secondary/private healthcare gatekeepers are not GPs. Although we believe data saturation was reached, our sample was derived from individuals returning the routine HMRN questionnaire about symptoms and help-seeking, so does not captured the views of people unable to do this, including those dying soon after diagnosis.

Strengths and weaknesses in relation to other studies

We were unable to identify any other qualitative studies exploring the experiences of patients with myeloma and only two specifically targeting lymphoma[36,37]. The latter were UK-based, and also included patient descriptions of challenging pathways and difficulties achieving diagnosis. Issues raised in these studies overlap those noted in the present work, indicating further progress in this area is still required. Furthermore, our study included people diagnosed within contemporary healthcare systems, after introduction of UK referral guidelines, targets and urgent diagnostic routes[4–6]; it is also the largest, and as noted, the only study including relatives.

For peer review only - http://bmjopen.bmj.com/site/about/guidelines.xhtml

Meaning of study

Our study clearly depicts the complexity of pathways to lymphoma and myeloma diagnosis. It clarifies the difficulties patients, relatives and HCPs have in identifying the underlying cause of what are often non-specific patterns of presentation (intermittent, vague, slowly progressive symptoms that are common in other illnesses), and some of the reasons likely to underpin: help-seeking, re-presentation and diagnostic delay[18–22]; and the lack of urgent hospital referrals for suspected cancer and high proportion of emergency presentations[23–25]. It also explains why interventions such as education campaigns targeting the (very broad) symptoms of these diseases, would lack impact. Interestingly, some patients reported symptoms present before and after diagnosis (e.g. nocturia: L24); whilst such symptoms were considered significant at the time, persistence after successful chemotherapy is likely to indicate the issue was unrelated to the DLBCL. This highlights the difficulty of distinguishing malignant from benign disease, particularly within a typically elderly population. Moving forward, it is important that this complexity is acknowledged within strategies to reduce time-to-diagnosis, including the new targets (more early stage cancer diagnoses) set out in the NHS Long Term Plan[8]. Importantly, the Rapid Diagnostic Centres introduced in this document target non-specific symptoms and could begin to address such difficulties, particularly if used in conjunction with rigorous safety netting in primary care[5,38], of which some evidence was seen in the current study. For members of the public, individual recognition of their own 'normal' is key, as well as having the confidence to pursue help-seeking for unexplained symptoms.

Unanswered questions and future research

Primary care HCPs have significant input into the care of patients later diagnosed with cancer, including those with emergency presentation[39]. To our knowledge, however, there are no qualitative studies exploring GP experiences of managing patients with lymphoma or myeloma. Nor is there any work linking primary and secondary healthcare data, although such studies would clarify the occurrence and timing of events between symptom onset and diagnosis and promote understanding of these complex pathways.

For peer review only - http://bmjopen.bmj.com/site/about/guidelines.xhtml

Table 1 Participant characteristics (n=55)

Diagnosis	Patients (Relatives) (n)	Males (Females) (n)	Age range (years)	Patient (P) ID
**Myeloma**	20 (17)	14 (6)	43-78	M1-20
**Lymphoma**	35 (15)	18 (17)	23-84	L1-35
**Non-Hodgkin lymphoma**				
Diffuse large B-cell	12 (7)	7 (5)	48-81	L2,4,8,9,20,21,24,27,29,31,32,35
Follicular	9 (2)	5 (4)	39-84	L1,3,6,10,12,13,18,22,26
Marginal zone	6 (1)	2 (4)	57-76	L5,16,25,28,30,33
Mantle cell	3 (3)	1 (2)	70-75	L11,15,34
**Hodgkin lymphoma**	5 (2)	3 (2)	23-56	L7,14,17,19,23

Table 2 Overview of interview schedule

Interviewees were asked to describe:

1. Initial and subsequent symptoms and health changes
2. Interpretation of symptoms and health changes
3. Response to symptoms between onset and diagnosis: actions; activities; events
4. Help-seeking episode(s) and factors influencing this
5. Where, when and with whom help-seeking occurred
6. Management by healthcare professionals (primary/secondary care)

For peer review only

Figure 1 Model of Pathways to Treatment[1]

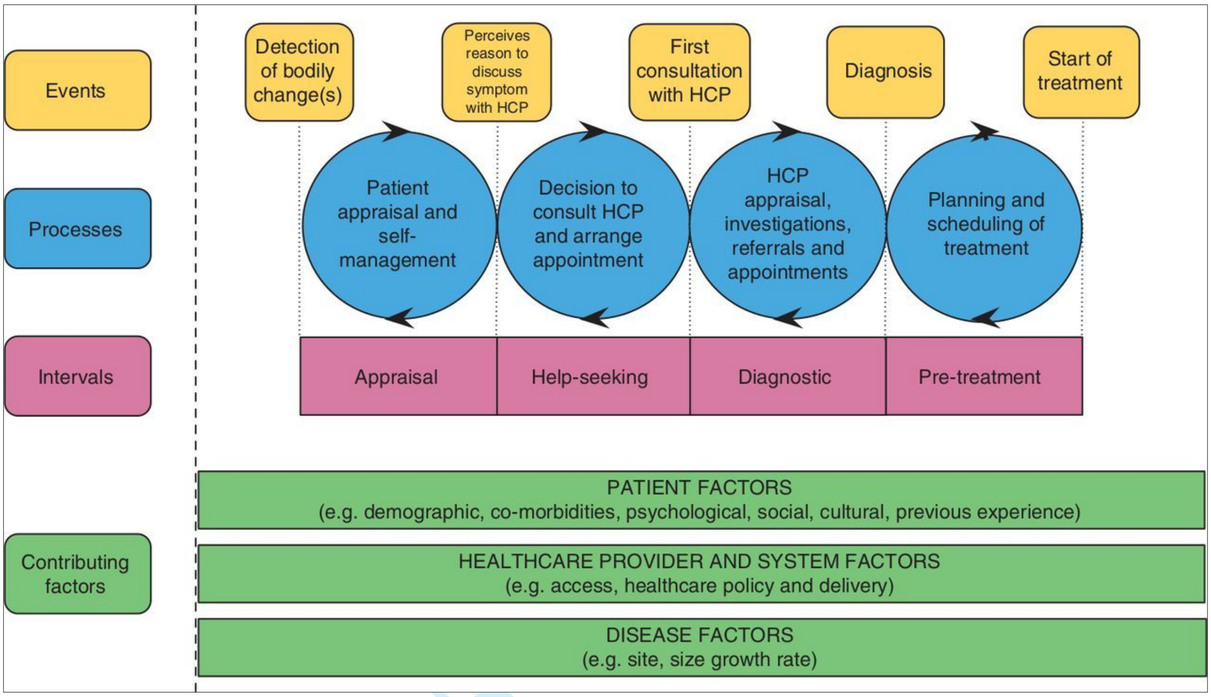

HCP: Health Care Provider

For peer review only - http://bmjopen.bmj.com/site/about/guidelines.xhtml

Figure 2: Summary of key themes and components

Theme 1: The appraisal interval

Appraisal and re-appraisal

Ongoing (intermittent or continuous), from symptom onset to diagnosis
Became more precise and formal over time (e.g. diary-documenting changes etc.)
Various explanations for symptoms considered
Information sought independently, from the internet, relatives, friends, colleagues etc.
Relatives involved and adapted their own behaviour the patient

Self-management

Occurred throughout pathway, not only during appraisal
Involved distraction techniques, use of aids, adapting everyday activities, self-medicating

Theme 2: Help-seeking and representation to diagnosis

All patients sought help from healthcare professionals (HCPs)
Could lead to identification of abnormality/suspected cancer and rapid investigation
Other conditions suspected: could be rapidly re-categorised or perpetuated
Broad health checks and general interventions requested
Only information the patient thought the GP would consider important provided
Impacted by advice from relatives, friends, colleagues etc.

Factors specific to re-presentation

Common and could occur on numerous occasions
Response to:

- worsening, progressive, new or alarming symptoms
- inability to perform 'normal' activities
- a growing feeling 'something' was wrong, but not knowing exactly what
- lack of progress towards diagnosis

Associated with negative investigations (especially blood tests)
Could be requested by the HCP (safety netting)
Requests became more specific (e.g. direct appraisal of specific sites, particular investigations)
Dependent on initial advice/diagnosis presented to HCP
Written evidence of symptom severity/persistence shown to HCP (e.g. diary)
Evidence from internet searching presented
Attend same GP or a different, specifically selected individual
Seek advice from various HCPs (dentists, physiotherapists, complementary therapists)
Hospital referral/admission requested
Emergency presentation (e.g. A&E: instigated by HCP, NHS helpline or patient etc.)
Involvement of relatives/others to advocate on behalf of the patient
Use of private interventions (e.g. physiotherapy, investigation, hospital referral)

For peer review only - http://bmjopen.bmj.com/site/about/guidelines.xhtml

Declarations

Funding: This study was funded by Cancer Research UK (National Awareness and Early Diagnosis Initiative: C38860/A13509); Bloodwise (15037); and the National Institute for Health Research: RP-PG-0613-20002.

Ethical approval: REC 04/01205/69 (HMRN); REC 12/YH/0149 (Qualitative study). The study was also registered with the UK Clinical Research Network Portfolio: UKCRN ID:11963.

Competing interests: The authors have declared no competing interests.

Authors contributions: DH, ER, AS, UM and RP devised the study, the methods and secured funding. RH and DH conducted interviews. RH analysed the data. UM and RP provided clinical input. DH prepared the draft manuscript. All authors edited and approved the final manuscript.

Data sharing: Ethical approvals do not permit data sharing. The corresponding author can be contacted for further information.

Acknowledgements: We are grateful to the patients and relatives who took part in this study and shared their experiences at a difficult time.

For peer review only

For peer review only - http://bmjopen.bmj.com/site/about/guidelines.xhtml

References

1. Walter, F., Webster, A., Scott, S. & Emery, J. The Andersen Model of Total Patient Delay: a systematic review of its application in cancer diagnosis. *J Health Serv Res Policy* **17**, 110–118 (2012).
2. Neal, R. D. *et al.* Is increased time to diagnosis and treatment in symptomatic cancer associated with poorer outcomes? Systematic review. *Br. J. Cancer* **112 Suppl 1**, S92-107 (2015).
3. Tørring, M. L. *et al.* Evidence of advanced stage colorectal cancer with longer diagnostic intervals: a pooled analysis of seven primary care cohorts comprising 11 720 patients in five countries. *Br. J. Cancer* **117**, 888–897 (2017).
4. NICE. *Referral Guidelines for Suspected Cancer*. (Department of Health, 2005).
5. NICE. *Suspected cancer: recognition and referral*. (2015).
6. Department of Health. *The NHS Cancer Plan: A plan for investment; A plan for reform*. (2000).
7. Neal, R. D. *et al.* Comparison of cancer diagnostic intervals before and after implementation of NICE guidelines: analysis of data from the UK General Practice Research Database. *Br. J. Cancer* **110**, 584–592 (2014).
8. NHS England. *NHS Long Term Plan*. (2019).
9. National Cancer Intelligence Network. *Cancer Incidence and Mortality By Cancer Network, UK, 2005*. (2008).
10. Westlake, S. Cancer incidence and mortality in the United Kingdom and constituent countries, 2004-06. *Health Stat Q* 56–62 (2009).
11. Jemal, A., Siegel, R., Xu, J. & Ward, E. Cancer statistics, 2010. *CA Cancer J Clin* **60**, 277–300 (2010).
12. Jemal, A. *et al.* Global cancer statistics. *CA Cancer J Clin* **61**, 69–90 (2011).

For peer review only

For peer review only - http://bmjopen.bmj.com/site/about/guidelines.xhtml

13. Swerdlow, S. *et al. WHO Classification of Tumours of Haematopoietic and Lymphoid Tissues*. (World Health Organization, 2017).

14. Smith, A. *et al.* Lymphoma incidence, survival and prevalence 2004-2014: sub-type analyses from the UK's Haematological Malignancy Research Network. *Br. J. Cancer* **112**, 1575–1584 (2015).

15. Smith, A. *et al.* Cohort Profile: The Haematological Malignancy Research Network (HMRN); a UK population-based patient cohort. *Int J Epidemiol* (2018). doi:10.1093/ije/dyy044

16. Howell, D. A. *et al.* Myeloma: Patient accounts of their pathways to diagnosis. *PLOS ONE* **13**, e0194788 (2018).

17. Howell, D. A. *et al.* Disease-related factors affecting timely lymphoma diagnosis: a qualitative study exploring patient experiences. *Br J Gen Pract* **69**, e134–e145 (2019).

18. Lyratzopoulos, G., Abel, G. A., McPhail, S., Neal, R. D. & Rubin, G. P. Measures of promptness of cancer diagnosis in primary care: secondary analysis of national audit data on patients with 18 common and rarer cancers. *British Journal of Cancer* **108**, 686–690 (2013).

19. Howell, D. A. *et al.* Time-to-diagnosis and symptoms of myeloma, lymphomas and leukaemias: a report from the Haematological Malignancy Research Network. *BMC Hematology* **13**, 1–9 (2013).

20. Keeble, S. *et al.* Variation in promptness of presentation among 10,297 patients subsequently diagnosed with one of 18 cancers: Evidence from a National Audit of Cancer Diagnosis in Primary Care. *Int. J. Cancer* **135**, 1220–1228 (2014).

21. Din, N. U. *et al.* Age and Gender Variations in Cancer Diagnostic Intervals in 15 Cancers: Analysis of Data from the UK Clinical Practice Research Datalink. *PLOS ONE* **10**, e0127717 (2015).

For peer review only

For peer review only - http://bmjopen.bmj.com/site/about/guidelines.xhtml

22. Koshiaris, C. *et al.* Quantifying intervals to diagnosis in myeloma: a systematic review and meta-analysis. *BMJ Open* **8**, e019758 (2018).

23. Elliss-Brookes, L. *et al.* Routes to diagnosis for cancer – determining the patient journey using multiple routine data sets. *British Journal of Cancer* **107**, 1220–1226 (2012).

24. Howell, D. *et al.* Multiple myeloma: routes to diagnosis, clinical characteristics and survival – findings from a UK population-based study. *Br J Haematol* **177**, 67–71 (2017).

25. Kane, E. *et al.* Emergency admission and survival from aggressive non-Hodgkin lymphoma: A report from the UK's population-based Haematological Malignancy Research Network. *European Journal of Cancer* **78**, 53–60 (2017).

26. Weller, D. *et al.* The Aarhus statement: improving design and reporting of studies on early cancer diagnosis. *Br J Cancer* **106**, 1262–1267 (2012).

27. Barbour, R. *Introducing Qualitative Research*. (Sage, 2019).

28. Tong, A., Sainsbury, P. & Craig, J. Consolidated criteria for reporting qualitative research (COREQ): a 32-item checklist for interviews and focus groups. *International Journal for Quality in Health Care* **19**, 349–357 (2007).

29. Sandelowski, M. Focus on Research Methods: Whatever Happened to Qualitative Description? *Res. Nurs. Health* **23**, 334–340 (2000).

30. Sandelowski, M. What's in a name? Qualitative description revisited. *Res. Nurs. Health* **33**, 77–84 (2010).

31. Roman, E. & Smith, A. The Haematological Malignancy Research Network: a UK population-based patient cohort. (2018).

32. Smith, A. *et al.* The Haematological Malignancy Research Network (HMRN): a new information strategy for population based epidemiology and health service research. *Br J Haematol* **148**, 739–753 (2010).

33. Glaser, B. & Strauss, A. *The Discovery of Grounded Theory*. (Aldine De Gruyter, 1967).

For peer review only

For peer review only - http://bmjopen.bmj.com/site/about/guidelines.xhtml

34. Charmaz, K. *Constructing grounded theory: A practical guide through qualitative analysis*. (Sage, 2006).

35. Lincoln YS & Guba EG. *Naturalistic Inquiry*. (Sage Publications, 1985).

36. Howell, D. A., Smith, A. G. & Roman, E. Help-seeking behaviour in patients with lymphoma. *Eur J Cancer Care (Engl)* **17**, 394–403 (2008).

37. Wall, C., Glenn, S. & Poole, H. Experiences prior to diagnosis of non-Hodgkin lymphoma: a phenomenological study. *J Adv Nurs* **67**, 2363–2372 (2011).

38. Safety netting. *Cancer Research UK* (2014). Available at: https://www.cancerresearchuk.org/health-professional/diagnosis/suspected-cancer-referral-best-practice/safety-netting. (Accessed: 11th July 2018)

39. Mitchell, E. D., Rubin, G., Merriman, L. & Macleod, U. The role of primary care in cancer diagnosis via emergency presentation: qualitative synthesis of significant event reports. *Br J Cancer* **112**, S50–S56 (2015).

For peer review only

Consolidated criteria for reporting qualitative studies (COREQ): 32-item checklist

Developed from:
Tong A, Sainsbury P, Craig J. Consolidated criteria for reporting qualitative research (COREQ): a 32-item checklist for interviews and focus groups. *International Journal for Quality in Health Care*. 2007. Volume 19, Number 6: pp. 349 – 357

YOU MUST PROVIDE A RESPONSE FOR ALL ITEMS. ENTER N/A IF NOT APPLICABLE

No. Item	Guide questions/description	Section/Page #
**Domain 1: Research team and reflexivity**		
Personal Characteristics		
1. Interviewer/facilitator	Which author/s conducted the interview or focus group?	DH & RH<br>P4 (Methods)
2. Credentials	What were the researcher's credentials? E.g. PhD, MD	DH: PhD, RGN<br>RH: MA
3. Occupation	What was their occupation at the time of the study?	DH: SRF<br>RH: RF
4. Gender	Was the researcher male or female?	Both female
5. Experience and training	What experience or training did the researcher have?	DH: Registered nurse, Researcher (20 years)<br>RH: Researcher (15 years)
Relationship with participants		
6. Relationship established	Was a relationship established prior to study commencement?	No, P4 (Methods)
7. Participant knowledge of the interviewer	What did the participants know about the researcher? e.g. personal goals, reasons for doing the research	Nothing, P4 (Methods)
8. Interviewer characteristics	What characteristics were reported about the interviewer/facilitator? e.g. Bias, assumptions, reasons and interests in the research topic	None
**Domain 2: study design**		
Theoretical framework		
9. Methodological orientation and Theory	What methodological orientation was stated to underpin the study? e.g. grounded theory, discourse analysis, ethnography, phenomenology, content analysis	P4 (Methods)
Participant selection		
10. Sampling	How were participants selected? e.g. purposive, convenience, consecutive, snowball	P4 (Methods)

For peer review only

11. Method of approach	How were participants approached? e.g. face-to-face, telephone, mail, email	P4 (Methods)
12. Sample size	How many participants were in the study?	P4 (Methods)
13. Non-participation	How many people refused to participate or dropped out? Reasons?	P4 (Methods)
Setting		
14. Setting of data collection	Where was the data collected? e.g. home, clinic, workplace	P4 (Methods)
15. Presence of non-participants	Was anyone else present besides the participants and researchers?	Relatives, who also participated, P4 (Methods)
16. Description of sample	What are the important characteristics of the sample? e.g. demographic data, date	Table 1, P12
Data collection		
17. Interview guide	Were questions, prompts, guides provided by the authors? Was it pilot tested?	Table 2, P12
18. Repeat interviews	Were repeat interviews carried out? If yes, how many?	N/A
19. Audio/visual recording	Did the research use audio or visual recording to collect the data?	P4 (Methods)
20. Field notes	Were field notes made during and/or after the interview or focus group?	P4 (Methods)
21. Duration	What was the duration of the interviews or focus group?	P4 (Methods)
22. Data saturation	Was data saturation discussed?	P4, P10 (Methods and Discussion))
23. Transcripts returned	Were transcripts returned to participants for comment and/or correction?	N/A
**Domain 3: analysis and findings**		
Data analysis		
24. Number of data coders	How many data coders coded the data?	P4 (Methods)
25. Description of the coding tree	Did authors provide a description of the coding tree?	N/A
26. Derivation of themes	Were themes identified in advance or derived from the data?	P4 (Methods)
27. Software	What software, if applicable, was used to manage the data?	N/A
28. Participant checking	Did participants provide feedback on the findings?	Corroborated by other patients (with similar experiences) at local support group.
Reporting		
29. Quotations presented	Were participant quotations presented to illustrate the themes/findings? Was each quotation identified? e.g. participant number	P4-10 (Results)

For peer review only

30. Data and findings consistent	Was there consistency between the data presented and the findings?	P10-P11 (Discussion)
31. Clarity of major themes	Were major themes clearly presented in the findings?	P4-P10 (Results) and P10-P11 (Discussion)
32. Clarity of minor themes	Is there a description of diverse cases or discussion of minor themes?	P4-P10 (Results) and P10-P11 (Discussion)

Once you have completed this checklist, please save a copy and upload it as part of your submission. When requested to do so as part of the upload process, please select the file type: *Checklist*. You will NOT be able to proceed with submission unless the checklist has been uploaded. Please DO NOT include this checklist as part of the main manuscript document. It must be uploaded as a separate file.

For peer review only

Standards for Reporting Qualitative Research (SRQR)*

http://www.equator-network.org/reporting-guidelines/srqr/

Title and abstract

	Page/line no(s).
**Title** - Concise description of the nature and topic of the study Identifying the study as qualitative or indicating the approach (e.g., ethnography, grounded theory) or data collection methods (e.g., interview, focus group) is recommended	P1
**Abstract** - Summary of key elements of the study using the abstract format of the intended publication; typically includes background, purpose, methods, results, and conclusions	P1

Introduction

**Problem formulation** - Description and significance of the problem/phenomenon studied; review of relevant theory and empirical work; problem statement	P3
**Purpose or research question** - Purpose of the study and specific objectives or questions	P3

Methods

**Qualitative approach and research paradigm** - Qualitative approach (e.g., ethnography, grounded theory, case study, phenomenology, narrative research) and guiding theory if appropriate; identifying the research paradigm (e.g., postpositivist, constructivist/ interpretivist) is also recommended; rationale**	P4
**Researcher characteristics and reflexivity** - Researchers' characteristics that may influence the research, including personal attributes, qualifications/experience, relationship with participants, assumptions, and/or presuppositions; potential or actual interaction between researchers' characteristics and the research questions, approach, methods, results, and/or transferability	P4
**Context** - Setting/site and salient contextual factors; rationale**	P4
**Sampling strategy** - How and why research participants, documents, or events were selected; criteria for deciding when no further sampling was necessary (e.g., sampling saturation); rationale**	P4
**Ethical issues pertaining to human subjects** - Documentation of approval by an appropriate ethics review board and participant consent, or explanation for lack thereof; other confidentiality and data security issues	P15
**Data collection methods** - Types of data collected; details of data collection procedures including (as appropriate) start and stop dates of data collection and analysis, iterative process, triangulation of sources/methods, and modification of procedures in response to evolving study findings; rationale**	P4

For peer review only - http://bmjopen.bmj.com/site/about/guidelines.xhtml

**Data collection instruments and technologies** - Description of instruments (e.g., interview guides, questionnaires) and devices (e.g., audio recorders) used for data collection; if/how the instrument(s) changed over the course of the study	P4, Table 2
**Units of study** - Number and relevant characteristics of participants, documents, or events included in the study; level of participation (could be reported in results)	P12, Table 1
**Data processing** - Methods for processing data prior to and during analysis, including transcription, data entry, data management and security, verification of data integrity, data coding, and anonymization/de-identification of excerpts	P4
**Data analysis** - Process by which inferences, themes, etc., were identified and developed, including the researchers involved in data analysis; usually references a specific paradigm or approach; rationale**	P4
**Techniques to enhance trustworthiness** - Techniques to enhance trustworthiness and credibility of data analysis (e.g., member checking, audit trail, triangulation); rationale**	P4

Results/findings

**Synthesis and interpretation** - Main findings (e.g., interpretations, inferences, and themes); might include development of a theory or model, or integration with prior research or theory	P4-10
**Links to empirical data** - Evidence (e.g., quotes, field notes, text excerpts, photographs) to substantiate analytic findings	P4-10

Discussion

**Integration with prior work, implications, transferability, and contribution(s) to the field** - Short summary of main findings; explanation of how findings and conclusions connect to, support, elaborate on, or challenge conclusions of earlier scholarship; discussion of scope of application/generalizability; identification of unique contribution(s) to scholarship in a discipline or field	P10-11
**Limitations** - Trustworthiness and limitations of findings	

Other

**Conflicts of interest** - Potential sources of influence or perceived influence on study conduct and conclusions; how these were managed	P15
**Funding** - Sources of funding and other support; role of funders in data collection, interpretation, and reporting	P15

*The authors created the SRQR by searching the literature to identify guidelines, reporting standards, and critical appraisal criteria for qualitative research; reviewing the reference lists of retrieved sources; and contacting experts to gain feedback. The SRQR aims to improve the transparency of all aspects of qualitative research by providing clear standards for reporting qualitative research.

For peer review only - http://bmjopen.bmj.com/site/about/guidelines.xhtml
For peer review only

**The rationale should briefly discuss the justification for choosing that theory, approach, method, or technique rather than other options available, the assumptions and limitations implicit in those choices, and how those choices influence study conclusions and transferability. As appropriate, the rationale for several items might be discussed together.

Reference:

O'Brien BC, Harris IB, Beckman TJ, Reed DA, Cook DA. **Standards for reporting qualitative research: a synthesis of recommendations.** *Academic Medicine*, Vol. 89, No. 9 / Sept 2014 DOI: 10.1097/ACM.0000000000000388

For peer review only

For peer review only - http://bmjopen.bmj.com/site/about/guidelines.xhtml

BMJ Open

‘Unpacking’ pathways to lymphoma and myeloma diagnosis: Do experiences align with the Model of Pathways to Treatment? Findings from a UK qualitative study with patients and relatives

Journal:	*BMJ Open*
Manuscript ID	bmjopen-2019-034244.R1
Article Type:	Original research
Date Submitted by the Author:	12-Dec-2019

Complete List of Authors:	Howell, Debra; University of York, Health Sciences<br>Hart, Ruth; University of Edinburgh, Usher Institute of Population Health Sciences and Informatics; University of York, Health Sciences<br>Smith, Alexandra; University of York, Health Sciences<br>Macleod, Una; Hull York Medical School<br>Patmore, Russell; Castle Hill Hospital, Queen's Centre for Oncology and Haematology<br>Roman, Eve; University of York, Health Sciences

**Primary Subject Heading**:	General practice / Family practice
Secondary Subject Heading:	Haematology (incl blood transfusion), Health policy, Health services research, Oncology, Qualitative research
Keywords:	Myeloma < HAEMATOLOGY, Lymphoma < HAEMATOLOGY, Diagnostic delay, Help-seeking, Patient pathways, QUALITATIVE RESEARCH

SCHOLARONE™
Manuscripts

I, the Submitting Author has the right to grant and does grant on behalf of all authors of the Work (as defined in the below author licence), an exclusive licence and/or a non-exclusive licence for contributions from authors who are: i) UK Crown employees; ii) where BMJ has agreed a CC-BY licence shall apply, and/or iii) in accordance with the terms applicable for US Federal Government officers or employees acting as part of their official duties; on a worldwide, perpetual, irrevocable, royalty-free basis to BMJ Publishing Group Ltd ("BMJ") its licensees and where the relevant Journal is co-owned by BMJ to the co-owners of the Journal, to publish the Work in this journal and any other BMJ products and to exploit all rights, as set out in our licence.

The Submitting Author accepts and understands that any supply made under these terms is made by BMJ to the Submitting Author unless you are acting as an employee on behalf of your employer or a postgraduate student of an affiliated institution which is paying any applicable article publishing charge ("APC") for Open Access articles. Where the Submitting Author wishes to make the Work available on an Open Access basis (and intends to pay the relevant APC), the terms of reuse of such Open Access shall be governed by a Creative Commons licence – details of these licences and which Creative Commons licence will apply to this Work are set out in our licence referred to above.

Other than as permitted in any relevant BMJ Author's Self Archiving Policies, I confirm this Work has not been accepted for publication elsewhere, is not being considered for publication elsewhere and does not duplicate material already published. I confirm all authors consent to publication of this Work and authorise the granting of this licence.

For peer review only

'Unpacking' pathways to lymphoma and myeloma diagnosis: Do experiences align with the Model of Pathways to Treatment? Findings from a UK qualitative study with patients and relatives

Authors: DA Howell[1], RI Hart[1#], AG Smith[1], U Macleod[2], R Patmore[3], E Roman[1]

Institutions:

[1]Epidemiology & Cancer Statistics Group, Department of Health Sciences, University of York, York, YO10 5DD, UK.

[2]The Hull York Medical School, University of Hull, Hull, HU6 7RX, UK.

[3]Department of Haematology, Queen's Centre for Oncology and Haematology, Castle Hill Hospital, Cottingham, Hull, HU16 5JQ, UK.

[#]Current address: Usher Institute of Population Health Sciences and Informatics, Old Medical School, University of Edinburgh, Edinburgh, EH8 9AG, UK.

[1]Corresponding author: debra.howell@york.ac.uk

Abstract

Objectives: To explore alignment of experiences before lymphoma and myeloma diagnosis with the appraisal, help-seeking and diagnostic intervals in the Model of Pathways to Treatment (MPT).

Design: A qualitative study using in-depth semi-structured interviews with patients and relatives. Interviews were transcribed verbatim, anonymised and analysed using qualitative description.

Setting: A UK population-based haematological malignancy patient-cohort.

Participants: Fifty-five patients (35 lymphoma, 20 myeloma: diagnosed 2014-2016) and 28 relatives participated, within around a year of the patient's diagnosis. Patients were selected from those in the cohort who had returned a questionnaire about their symptoms and help-seeking, and consented to contact for further research. Sampling was purposive, to achieve maximum variation in age, sex and time-to-diagnosis.

Results: Participants described time from symptom-onset to diagnosis as ranging from several weeks to years. Pathways largely aligned with MPT components and help-seeking could lead to the rapid investigations and identification of abnormalities. However, symptoms could be vague and/or inadvertently interpreted as other conditions, which if perpetuated, could cause diagnostic delay. The latter was associated with chaotic pathways, with activities rarely occurring only once or in a linear sequence. Rather, intermittent or ongoing processes were described, moving forward and backwards through intervals. This is 'unpacked' within five themes: 1) appraisal and re-appraisal; 2) patient-initiated self-management/treatment; 3) initial help-seeking; 4) re-presentation; and 5)

For peer review only - http://bmjopen.bmj.com/site/about/guidelines.xhtml

patient-initiated actions, decisions and emotions during re-presentation. Within these themes, various healthcare professionals were consulted, often many times, as symptoms persisted/progressed. Input from family/friends was described as substantial, as was the extent to which information seeking occurred.

Conclusion: Lymphoma and myeloma pathways align with the MPT, but do not fully capture the repetition and complexity described. Time-to-diagnosis was often prolonged, despite best efforts of patients, relatives and healthcare professionals. The impact of NHS England's Multi-diagnostic Disciplinary Centres on time-to-diagnosis remains to be seen.

For peer review only

Strengths and limitations of this study

- In-depth interviews generated detailed data about complex experiences before diagnosis.
- The views of patients and relatives are captured, within contemporary healthcare systems.
- Maximum variation sampling ensured findings are largely transferable to similar settings.
- Patients were interviewed within a year of diagnosis and asked to use diaries to promote recall.
- As the sample was derived from individuals returning a questionnaire about their symptoms and help-seeking, the views of people unable to do this are not captured.

Keywords: Myeloma, lymphoma, diagnostic delay, help-seeking, patient pathways, qualitative research

For peer review only

1.0 Introduction

Early diagnosis of symptomatic cancer can lead to improved quality of life and survival[1,2], and has been a long-term UK policy priority, promoted via GP referral guidelines and waiting-time targets[3–5]. The impact of such measures has been variable, however, with substantial reductions in time-to-diagnosis seen for some cancers, but limited benefits for others; and some actually seeing increases[6]. Further targets and initiatives introduced in the recent NHS Long Term Plan[7], as well as a broader range of potential pathways to diagnosis[8,9], confirm this as an area that continues to be clinically relevant, with early diagnosis still considered key to improving UK cancer survival.

Arising in blood and lymph forming tissues, haematological malignancies (leukaemia, lymphoma and myeloma) are collectively the fourth most common cancer among men (after prostate, lung and bowel) and women (after breast, lung and bowel) in economically developed regions of the world[10–13]. With diverse pathways and treatments, the World Health Organization currently recognizes over 100 heterogeneous haematological malignancy subtypes, which display a range of behaviours and outcomes[14]. Myeloma, for example, generally presents in the bones and often requires intermittent treatment throughout a remitting-relapsing pathway, that is more typical of chronic disease than cancer. Non-Hodgkin lymphoma and Hodgkin lymphoma each have further subtypes, arise in various nodal/extra-nodal site(s) and may be indolent or aggressive. Myeloma and indolent lymphomas (e.g. follicular lymphoma) are considered incurable at diagnosis, but can often be controlled for long periods of time. Aggressive lymphomas (e.g. diffuse large B-cell lymphomas) are potentially curable with chemotherapy, but many patients still die from these disease.

Patterns of disease onset and time-to-diagnosis of lymphoma and myeloma are varied. For example, patients may be asymptomatic and diagnosed incidentally or they may have multiple and/or severe symptoms, that can be diverse and similar to other self-limiting conditions (e.g. fatigue, bone pain, swollen glands) to which they are often initially attributed or 'normalised'; symptoms may also be vague and non-specific in onset or intermittent[15–17]. Importantly, time-to-diagnosis can be prolonged and associated with multiple GP consultations before hospital referral[18–23], myeloma being one of very few cancers that has seen increases in time-to-diagnosis in recent years, despite policy initiatives[6]. Emergency presentation is comparatively common in these cancers, a route linked to advanced disease, more complications and poorer survival than other pathways[24–26]. Conversely, fewer two-week wait referrals are made to secondary care and more routine GP referrals than for other malignancies, suggesting cancer is not suspected at the time of hospital referral.

In the UK, a contemporary schematic template (known as the Model of Pathways to Treatment: MPT, Figure 1)[27] is recommended for mapping and examining pathways to cancer diagnosis, along with a checklist to ensure consistent definitions, terminology, methods, data collection and reporting between studies[28]. The MPT comprises a series of intervals beginning with appraisal (patient appraisal of symptoms and self-management); followed by help-seeking (decision to consult a healthcare professional (HCP), and make and attend an appointment); the diagnostic interval (HCP appraisal, investigation, and referral); and the pre-treatment interval (diagnosis to the start of treatment). Each interval is impacted by contributing factors, including patient actions, healthcare systems and disease characteristics. The MPT authors acknowledge that the intervals they describe may require 'unpacking' by cancer-type[27]. The overarching aim of the current paper is to address this by 'unpacking' the MPT, based on the experiences described by patients with lymphoma and myeloma and their relatives, in the time leading to diagnosis.

For peer review only - http://bmjopen.bmj.com/site/about/guidelines.xhtml

2.0 Methods

Given the difficulties described above in achieving lymphoma and myeloma diagnosis, a mixed methods study was set-up to explore the experiences of patients (and relatives) before diagnosis, which has, to date, resulted in several publications using data from medical records and qualitative interviews[16,17,25]. The current paper is based on the qualitative study phase, the methods of which are outlined below in accordance with the consolidated criteria for reporting qualitative studies (COREQ)[29]. Qualitative data are known to be well suited to investigating how people make sense of their situation, and how and why they make particular decisions[30]. In this study, our theoretical approach adhered to the principles of qualitative description[31,32], an applied method facilitating the production of concrete, low inference descriptions with practical relevance. The MPT[27] was used as a framework from which to examine activities within the appraisal, help-seeking and diagnostic intervals, as this is where the most significant delays are considered to occur[17].

The qualitative study examining diagnostic pathways was based within the UK's Haematological Malignancy Research Network (HMRN: www.hmrn.org), detailed methods of which have been published elsewhere[33,34]. Briefly, HMRN is a specialist population-based cohort which was initiated in 2004. It tracks entire treatment pathways and collects data on all patients newly diagnosed with haematological malignancies across 14 hospitals (catchment population ~4 million) in the North of England (Yorkshire and Humberside). In addition to clinical data collection, HMRN patients are routinely asked to complete a questionnaire about their symptoms and help-seeking.

Potential interviewees for the qualitative study were derived from patients completing the routine questionnaire in the HMRN area, who were aged ≥18 years, and within around 12 months of diagnosis. Sampling was purposive and designed to achieve maximum variation in age, sex and time-to-diagnosis. Ninety-two people were posted study material and invited to contact the research team if they wanted to participate; they were also invited to ask a relative/carer to contribute to the interview if they wished. Fifty-five patients (diagnosed 2014-2016; 20 with myeloma; 35 with lymphoma) and 28 relatives were interviewed, with data collection continuing until saturation. An overview of patient characteristics and the duration of intervals can be found in Table 1, with further information provided in the Supplementary Table, S1.

For peer review only - http://bmjopen.bmj.com/site/about/guidelines.xhtml

Table 1 Overview of participant characteristics (n=55)

Diagnosis	Patients (Relatives[1])	Total females	Total males	Median age Years (range)	Duration of int
					Appraisal/help-seeking[2]
Non-Hodgkin lymphoma[4]					
• *Diffuse large B-cell*	12 (7)	5	7	64 (48-81)	0.5-13
• *Follicular lymphoma*	9 (3)	4	5	63 (39-84)	0.5-12
• *Marginal zone*	6 (1)	4	2	62 (57-76)	0.5-11
• *Mantle cell*	3 (3)	2	1	71 (70-75)	1-6
Hodgkin lymphoma[4]	5 (2)	2	3	36 (23-56)	0.5-12
Myeloma[4]	20 (12)	14	6	68 (43-78)	0.5-7
**Total**	**55 (28)**	**31**	**24**	**64 (23-84)**	**0.5-13**

[1]Spouses, partners, children; [2]Initial symptom/health change to first help-seeking (from information estimated by patients completing the routine self-reported HMRN questionnaire). [3]First help-seeking to diagnosis (start point also estimated from the self-reported HMRN questionnaire); [3]Initial symptom/health change to diagnosis.

For peer review only

For peer review only - http://bmjopen.bmj.com/site/about/guidelines.xhtml

Interviews (conducted November 2015-May 2016 by RH and DH, both of whom were previously unknown to participants), were semi-structured and guided by a schedule, developed from existing literature, as well as clinical advice from within the study team (Table 2). With written consent, patients were asked to describe their pathways from first symptom(s) or health change(s) to diagnosis. Most interviews were carried out face-to-face in the patients home, although several were conducted in hospital or university settings, and a minority occurred by telephone. Interviews lasted around 45 minutes and were audio-recorded, transcribed, anonymised and checked, with field-notes used to confirm accuracy and support data analysis.

Table 2 Overview of interview schedule
Interviewees were asked to describe:
**1. Initial and subsequent symptoms and health changes**
**2. Interpretation of symptoms and health changes**
**3. Response to symptoms between onset and diagnosis: actions; activities; events**
**4. Help-seeking episode(s) and factors influencing this**
**5. Where, when and with whom help-seeking occurred**
**6. Management by healthcare professionals (primary/secondary care)**

Analysis was iterative, running alongside and informing data collection (RH, DH). After review and familiarisation with transcripts, narrative summaries were produced from the data and pathways were mapped-out visually and shared with the research team (ER, AS). Several rounds of coding were undertaken, focusing on different aspects of the data, including activities and events occurring within intervals defined in the MPT. Constant comparison (codes with codes, codes with data, codes with theory) and memo-ing[35] supported refinement of codes and identification of patterns and relationships. Codes and emerging themes were discussed by the team and refined until consensus was reached. Input from relatives was analysed alongside that of the patient, using the same methods.

Patient and Public Involvement (PPI)

PPI is integral within HMRN, and takes place via a dedicated Partnership, overseen by a lay-committee. Patients from the Partnership were involved in identifying the research questions underpinning the study during several focus group meetings, where they highlighted the difficulties they had experienced getting diagnosed; they were also involved in study design via our funding application. Furthermore, patients routinely assist in the dissemination of all HMRN findings, which also occurs via our lay-website: www.yhhn.org.

3.0 Findings

Participants described time from symptom-onset to diagnosis as ranging from several weeks to numerous years. Within this disparate time-frame, pathways largely aligned with MPT, although experiences were more chaotic and did not always fit this model's distinct intervals or linear trajectory. This is 'unpacked' within five themes: 1) appraisal and re-appraisal; 2) patient-initiated

For peer review only - http://bmjopen.bmj.com/site/about/guidelines.xhtml

self-management/treatment; 3) initial help-seeking; 4) re-presentation; and 5) patient-initiated actions, decisions and emotions during re-presentation. Each theme is described below using illustrative verbatim quotes and summarised in Figure 2.

3.1 Appraisal and re-appraisal

Patient appraisal of symptoms and health changes was described as ongoing by participants, with observation and monitoring occurring periodically or continuously from first symptom to diagnosis. When 'normal' health was not regained, appraisal was said to become more deliberate and rigorous. It involved, for example, checking and comparing affected and non-affected sites: *'My mind was going, "If you can feel* (a lump)*...both sides, then it's normal. If you can feel it on one side, then that's something to worry about". So I tried, I felt the other side, and again…'* [Lymphoma patient 06: L06]. This was said to take place repeatedly over time: *'I couldn't decide whether my skin was a different colour…I'd look at it and think, "It looks strange", and then I'd look again, and it didn't'* [L03].

Interviewees described re-appraising persistent, progressive or new symptoms, which could be perceived to be related, or unrelated, to the initial problem. Unremitting symptoms sometimes led to more precise and formal appraisal, including documentation of health changes, pain, medication and weight: '*I kept a note, like week-by-week almost'…*[L25]. A key element of appraisal involved patients and relatives considering numerous explanations for their symptoms over time. This included 'normalisation' of symptoms by attributing these to life-course issues (e.g. age or menopause related) or events (decorating), or self-limiting conditions: '*Could I have ME?'* [L05]; *'Maybe it was an ulcer'* [L26]; *'I was just thinking, "actually, is this becoming a mental health issue? Is it* (the) *first signs of dementia?"'* [Relative of myeloma patient 20: M20].

Interviewees described information seeking from various sources during appraisal/re-appraisal, particularly for ongoing symptoms; an issue not wholly acknowledged in the MPT. One myeloma patient consulted relatives about their family history (osteoporosis) as a potential explanation for symptoms. Others described questioning medically experienced friends, colleagues or acquaintances: *'There's a doctor…lives further down the lane…I said* [to him] *"I don't really like asking but…"'* [L03]. Many explored symptoms via the internet: *'I'd looked at all sorts of problems I thought* (patient) *might be having with bones"'* [Relative, M20]. Internet searching was more effective when symptoms were typical of myeloma or lymphoma: *'I think I put in something like "itchy skin, night sweats, lump in neck"; Hodgkin's lymphoma came up, through a few different clicks'* [L14]. This could lead to rapid help-seeking/re-presentation: *'had I not seen* (information)*, I'm not convinced I would have said anything to the doctor, but because I'd seen it and I thought, "Crying out loud…", I was like, "We've got to do something…someone needs to put all these pieces of the puzzle together"'* [Wife, L14]. It could also lead to emergency presentation: '*the more I read* (about lump)*, the more I was scared, and I just went to A&E…'"* [L23]; or denial: *'Well, when I started reading about cancer I just switched it off, because I thought, "No, no, that's not right"'* [L05].

Considerable involvement of relatives, friends and colleagues in appraisal/re-appraisal was described, though not wholly depicted in the MPT: *I just looked at his tummy and I thought, "that's an odd shape, that", and it just flashed through my mind and I thought "That could be a tumour"'* [Relative: L20]. Some relatives said they adapted their own behaviour to accord with the patient, often over long time-periods: '(Patient's) *height loss, was gradual, at first, so, I noticed…I stopped*

For peer review only - http://bmjopen.bmj.com/site/about/guidelines.xhtml

buying shoes with heels, because I was getting to be, you know, too tall (for him) *when I was wearing shoes with heels…That had probably been going on…maybe a couple of years'* [Relative: M18]; or they began to discuss the patient's symptoms and health changes between themselves: *'We knew there was something wrong…my daughter actually said, "You know, dad's not right at all, is he?"'* [Relative: M02].

3.2 Patient initiated self-management/treatment

Self-management was not solely associated with the appraisal interval as portrayed in the MPT, but was described as occurring throughout the pathway, particularly if patients or HCPs had normalised initial symptoms to self-limiting or life-course issues (e.g. age, menopause): *'we're the kind of people who go to the doctor as a last resort, and we'd tried the obvious things ourselves, you know* (lists remedies for indigestion)*, anything you think might ease it. But it didn't…'* [Relative: L20].

Some patients said they used distraction techniques for relief, often long-term: '*I wake up because my tummy's hurting, and then I get up and start the day, and then I try and fill my day with things, so that I don't think about it…and, that was how it went and, we're probably talking a couple of years of that…just putting up with these different things'* [L26]. Others reduced or stopped activities viewed to cause/aggravate problems: *'You'd stopped driving for about two months (before diagnosis), because you were in…pain'* [Relative: M03]; [Interviewer: 'You gave up work 18 months ago…?'] '*Yeah…it were getting a little bit difficult for me, and I didn't want to embarrass myself'* [L16].

People described using aids over long time-periods: '*I used to go to work with that belt* (meant for weight-lifting)*, you know, on my back'* [M15]. Others used sticks, wheelchairs or riser-chairs to remain mobile. Lifestyle changes were made: *'I've had grumbling problems… probably at least two years…the kind…you treat yourself, with indigestion medicine, drinking water, trying to eat well, going for exercise, all those things that you think "This'll sort me out. I'm just getting a bit older and I need to look after myself"* [L26]. Some took supplements *'we were having Complan drinks with whole milk…protein drinks, powders, anything to get something into his system…the weight back'* [Relative: L20]; or self-medicated with analgesics, anti-inflammatories, anti-histamines and gargles.

Long-term strategies were reported to manage debilitating tiredness, including napping or sleeping more: '*I were feeling tired all the time…I used to eat my sandwiches before my lunch…as I went round, and then as I stopped the van, I used to turn the ignition off, and lay on the seat, and go to sleep'* [L16]; *'I tended to sleep a lot. I'd come home for lunch at 12 o'clock…fall asleep in the chair… almost every day…'* [M12]. *And then you'd come home and fall asleep, and you'd wake up and have tea, then fall asleep again, and then wake up for a couple of hours watching telly, and then go to bed*' [Relative: M12].

Some people said they chose to pay for specific treatments, with mixed results: *'*(physiotherapy) *made matters worse'* [M18]; '*eventually, he were in that much pain, he couldn't go...* (to physiotherapy)*'* [Wife: M15]; '*I went to an osteopath…which did help'* [M12]; An '*herbalist' and an 'allergist'* were also consulted; and one person considered '*colonic irrigation*'.

3.3 Initial help-seeking

Concurring with the MPT, all patients described help-seeking for symptoms and attended their GP at some point. The decision to seek help and the timing of this event was often said to be influenced by relatives, friends or colleagues, who could encourage help-seeking: '*My mum and friends* (were)

For peer review only - http://bmjopen.bmj.com/site/about/guidelines.xhtml

saying, "You…really ought to go to the doctors"' [M05]; or indeed discourage it: *'I had these lumps on my neck and I, I didn't take any notice of them…I said to a friend and she said…"Oh it's nothing, everybody gets lumps"'* [L15].

A minority said initial help-seeking led to the rapid identification of abnormality/suspected cancer, which was quickly investigated and diagnosed: '*The doctor…was lovely and she examined* (patient)…*and said "I can feel something, in there, and she booked him in…for a CT scan…endoscopy, yeah, and a blood test…on the scan they saw, there was a mass'* [Relative: L20]; '(GP) *did a very thorough check…he's always been very thorough...physical and, yeah, mental if you like…I've always found him absolutely superb.* (He) *listens not just to what you say, but what you don't say and, and sort of, reads the signals;* [L01]; (GP) *decided it was something serious, and then you were sent for a scan…you had a biopsy, and it all happened very quickly*' [Relative: L01].

Initial help-seeking could include requests for broad health-checks*: 'I just felt, okay I'll go to the doctor's and…*(say), *"Well, I want an MOT…I seem a bit tired"…and it was a locum, and I explained my symptoms, and he sort of said, "Well, they seem a bit of an odd set of symptoms, so we'll give you…full blood tests", and thank goodness he did'* [M05]; *'I did sort of ask for like a 'well woman' check, because I didn't in my mind feel like I was right'* [L17]. General interventions were also sought: *'I was just really feeling fed up, because I was so tired…I went for a tonic, I said, "Can you give me just something to pick me up?"'* [L34]. Alternatively, other conditions were initially suspected, particularly if these conditions had symptoms that were also associated with myeloma or lymphoma: *'I noticed a swelling at the top of my groin…*(GP) *said "that feels like a, a hernia to me…I'll sort you out an appointment to get you referred"'* [L06].

3.4 Re-presentation

As initial help-seeking only rarely led to diagnosis, re-presentation was extremely common. This was said to occur quickly with increasingly alarming symptoms: *'(I) went to the bank, and wet myself… and I thought, "Oh my God… what's going on here?" And I went* (back) *to the GP'* [L09]; *'the sickness…he started bringing his evening meal up, and unable to sleep at night, the pressure on his tummy…he'd be walking the floor, you know, just generally, obviously something very wrong* [L20].' It could be delayed if the patient was told resolution may take time: *I saw two* (GPs)*…* [Interviewer: Who said?] *'Give it time'* [M13]; or if symptoms were intermittent: *'he'd be ok for a while, and then, you know, maybe a few nights he'd be sick'* [Relative: L20]; *'I carried on working, as you do…I didn't feel too bad really, some of the time'* [L20].

Alternatively, repeat GP consultations could be delayed, particularly if initial/differential diagnoses were perpetuated: *'I noticed, like, a little pea-sized lump in my leg…my right calf…I thought… "Probably me varicose veins". I didn't rush, I went to see the doctor, regarding something else, and I just happened to mention to him about it… he had a feel of it, and he says "right, we'll send you off to have and ultrasound", you know, get it checked out. I asked* (radiographer)*…"Is it a varicose vein?" She says "yes". 'I got on with my life, I just carried on. I thought well fair enough, you know, its varicose veins…I never bothered when I started getting other lumps in my leg, I thought, "well it's all just part of it".* (The following year) *this lump was steadily growing…I started getting other lumps…the lumps joined together…starting to turn red and poke out of my skin...It just sort of exploded sort of thing, you know, it was frightening how fast it was growing…I went to see the doctor again'* [L31].

Re-presentation often occurred on multiple occasions: *'I went back probably about three times between February and August…* [M13]; '*all that time, just going backwards and forwards to the*

For peer review only - http://bmjopen.bmj.com/site/about/guidelines.xhtml

doctors' [Relative: L24]. Sometimes this was associated with ongoing normal investigations: '*I just felt so tired. I'd just no energy, you know, and the daft part is, I think I'd three lots of blood tests done as well, to try and find out, you know, and yet none of 'em showed anything to do with the lymphoma'* [L31]; '*The blood test revealed nothing, did it, and neither did the camera in the stomach*' [L20]. Where abnormal blood tests were noted, rapid hospital referral was described: '*I'd had about four or five different blood tests, and it were all coming back negative*' [M15]…'*til these blood tests did come back abnormal, all of a sudden, so that's when they sent him up to haematology* [Relative: M15]; '(GP) *immediately, more or less said, I'm sending you into hospital. You're anaemic*' [L24].

Repeat consultations sometimes occurred at the request of HCPs: '(GP) *said to me if there are ever any changes with the lumps to inform him*' [M12]. This was sometimes associated with HCPs expressing frustration about their inability to explain symptoms: '(GP) *said: "come back in four weeks…I'm getting me books out…I don't understand what the symptoms are pointing me to*" [L24, paraphrasing GP]. Interestingly, this patient's main concern ('*night urination…combined with the loss of weight, that was what really got me, erm, worried*'), remained unresolved after successful treatment, and at this point was considered unrelated, with urology review awaited.

Re-presentation also occurred due to a growing conviction something was really wrong: *'Normally I'm not somebody who goes to* (the) *doctors…it was just a feeling that something definitely wasn't right. Despite getting the first opinion'* [L01]; *My mobility was shocking by then…I was having great difficulty in doing anything, literally and I knew then that there was something…*' [M03]; '*I weren't going to take "no" for an answer… because I knew there was something'* [L34]. Doubt about an earlier explanation was often key: '*I was convinced there was something wrong with me, other than osteoporosis, because I thought "I shouldn't feel, this unwell, with osteoporosis."'* [M14].

HCPs directed a number of patients towards emergency (re)presentation, either via A&E or a direct ward admission, often for worsening symptoms and to gain rapid access to healthcare. '*I saw the nurse, and she said, "Look, you know, go to A&E and let them have a look at it, so that's what we did'* [L31]. Symptom complexity could result in consultation with multiple specialists even via this route, with the same patient being assessed by the A&E team, then a physiotherapist, vascular surgeon and plastic surgeon, before being told "it's a tumour" [L31].

3.5 Patient-initiated actions, decisions and emotions during re-presentation

Some interviewees described requesting more specific input from their HCP at re-presentation, including direct appraisal: '*you need to see my neck again'* [L14]; '*have a look while I'm standing up*' [L34]; '*I said to* (GP)*…"Can you actually measure me?" which she did…she indicated… that I'd lost about two inches'* [M14]; and particular investigations: '*I said, "Look, there's something wrong here. I need an X-ray"'* [M13]; *'I said "I'm not picking up" and she agreed* (to blood tests)*'* [L05].

Evidence (e.g. diaries) was often produced by patients at re-presentation to demonstrate the extent and severity of symptoms: *I thought, "*(GP's) *probably thinking I'm just a hypochondriac". But when I got the evidence of the weight that I was losing, I didn't feel that…*(GP) *could turn a blind eye to that.'* [L25]. New information from re-appraisal (symptoms, mismatch between symptoms and preliminary diagnoses, and test results) or internet searching was also said to be passed on to HCPs: *'I said, "I think I've got lymphoma…I seem to have these lumps that haven't gone down at all…and I think now I'm getting, it's either anxiety, or I'm now getting B-symptoms, which is night sweats…"'* [L06]. This could prompt action: *'I went back to the GP and said, "Well,* (ENT) *say it's TB or lymphoma", and the*

For peer review only - http://bmjopen.bmj.com/site/about/guidelines.xhtml

GP said, "Well, I don't think it's lymphoma, but, if they're saying TB, I'll send you for an X-ray"' [L07]; but not always: *'I'd been in pain for a long time, and I actually said, "I think I've got cancer" and (Dr) said to me, "You don't look like* somebody *who's got cancer"'* [M14]; *'*(GP) *said, "No, no, you haven't…lymphoma, old people get lymphoma. You're* (under 40)*…don't even think about that"'* [L06]. Patients reported chasing-up information to give to HCPs (e.g. results): *'I rang* (surgery)*, three times a week, every week, to see if they'd had a reply'* [Relative: L11].

Others blamed themselves for their delayed diagnosis, saying they only gave HCPs partial information about their symptoms: *'maybe I wasn't forthcoming with the information'* [M03]; either purposely (only reporting symptoms they thought the HCP would considered important) or inadvertently: *'I didn't mention my general apathy, because I just thought it was just apathy to be honest'* [M12]*; 'many's the time I've come out thinking, "Oh damn, I didn't mention that"' [L24].*

A few patients described feeling obliged to '*stick with*' the first GP consulted: '*he knew the situation, and I kind of felt like, it'd have been going behind his back to go and see another GP'* [L07]. Others did not: *'The very first doctor I saw. I saw him twice and then I knew after that I wasn't going to ask for that particular doctor'* [M14]. Some reported re-presenting to HCPs they knew and trusted, were more experience, or just different: *'We would sometimes just ask for a different one, just to get a different opinion'* [L34]. Decisions were also made to consult various other specialists: '*I'd started to suffer with mouth ulcers, and…I actually went to my dentist'* [L30]; and advice was sought at unrelated hospital appointments: *'I went to see my Crohn's surgeon… my normal check-up, and I mentioned (the lump) to him, and he's the one really who pushed it forward fast…'* [L32].

Lack of progress towards diagnosis could lead patients and relatives to ask their HCP to refer/admit them to hospital, which could be refused: *'"Oh no, she doesn't need to go to hospital"'* [Relative paraphrasing HCP, L35]. It also resulted in distress, anxiety and frustration: *''I was getting towards my wits' end, because I knew that there was something wrong, and I just felt as if* (HCPs) *weren't taking me seriously'* [L06]. Occasionally, this resulted in emotional outbursts: *'The doctor said, "Have some antibiotics"… and we broke down and were just like, "This isn't right". So he called the hospital and got* (Patient) *into hospital'* [Relative, L14]; *'They thought it was anxiety, because I'd got myself in a state, and I was in a tearful mess at the doctor's, because of feeling ill and they're not getting anywhere…So she just thought it was an anxiety issue. It was like turned from one thing to something else'* [L17].

Patients said their relatives often attended later GP appointments with them to advocate on their behalf: *'you become increasingly dependent on somebody to go with you…for reassurance and support'* [M14]; *'We made and appointment…we had to insist* (patient) *needed to see someone, and we went together, and we said, "we need help here"'* [L20]; *'"I'm not waiting for this appointment", I said, "because (Husband, the patient) can't wait". I said, "I want him to see* (haematologist)*, as soon as!" I said, "I know you can do it, because I worked in the health-service" so she said, "Yes, okay". Otherwise, I honestly thought he could sit in the chair and just go* (die)*. He was so ill'* [Relative: L11].

Some patients and relatives recalled emergency presentation to hospital via A&E, which they initiated themselves: *'I was getting so desperate…I knew something was wrong, and I felt that nobody was really listening to what I was saying. I did actually go to A&E a couple of times. Well, once under our own steam, and once we rang 111, and they said "Go to A&E"'* [M14]. Several people also mentioned eventually seeking private investigations and/or hospital referral, which they considered key to diagnosis. Access to private healthcare was said to be rapid: '*it bought me time'*

For peer review only - http://bmjopen.bmj.com/site/about/guidelines.xhtml

[L13]. Where GPs knew patients had private insurance, they were reported to suggest referral *'I went backwards and forwards to my GP, and I'm covered by my husband's private hospital thing, so* (GP) *suggested that I went to see a consultant back specialist*' [L09]. More commonly, patients portrayed private care as an extreme but necessary measure: *'the timeframe for me was…it was too much, so I said "I want a private referral"'* [M12].

4.0 Discussion

Principal findings

Pathways to lymphoma and myeloma diagnosis largely aligned with the MPT[27]. However, further 'unpacking' confirmed that trajectories were often much more complex and chaotic than depicted in the model, with activities rarely described as occurring on a single occasion or in a linear sequence. Rather, intermittent or ongoing processes were described, that moved forward through intervals (e.g. appraisal to help-seeking), but also back to the start of the pathway, so that repeat/continuous re-appraisal occurred, with multiple re-presentations to HCPs, which could cause anxiety and distress. Symptoms were 'normalised'[15] and self-management occurred across pathways, not only within the appraisal interval. Patients and relatives were also active within the diagnostic interval. Input from family and friends was substantial in all intervals, as was the extent to which independent information seeking occurred. Disease, patient and HCP factors (as presented in the MPT model), were found to influence each part of the pathway, with symptoms and events at one time-point (e.g. GP reassurance about symptoms or a normal investigation) influencing later actions and time-scales (e.g. delayed patient re-presentation; delayed HCP referral to hospital). Similar experiences were reported by patients with myeloma and lymphoma (although more patients with myeloma were referred to hospital by the GP after an abnormal blood test); and various HCPs were consulted throughout the diagnostic interval.

Strengths and weaknesses

This is the only qualitative study exploring pathways to diagnosis of lymphoma and myeloma in the context of the MPT, whilst taking an overview that tries to capture the interactions between people (patients, relatives and HCPs) and the events/activities taking place throughout the pathway. Our study included people diagnosed within contemporary healthcare systems, and after introduction of UK referral guidelines, targets and urgent diagnostic routes[3–5]. The sample is comparatively large (55 patients; 28 relatives) and in-depth discussions ensured findings were based on personal experiences and perceptions. Inclusion of relatives is a unique feature and a particular strength as these individuals (spouses, partners and children), were able to contribute their own views of the pathway and their involvement in events. Participation from relatives ranged from them taking a minor role (for example reminding the patient about dates and events), to having significant input in describing the pathway and their own actions and emotions. Interviews were conducted within a year of diagnosis and the use of letters, calendars and diaries was encouraged to promote recall. Although generalisability is not an overarching aim of qualitative research, maximum sample variation increased the transferability[36] of findings to places with similar healthcare systems. Experiences may, however, differ in countries without universal provision, or where secondary/private healthcare gatekeepers are not GPs. Although we believe data saturation was reached, our sample was derived from individuals returning the routine HMRN questionnaire about symptoms and help-seeking, so did not capture the views of people unable to do this, including

For peer review only - http://bmjopen.bmj.com/site/about/guidelines.xhtml

those dying soon after diagnosis, who may have had more advanced stage disease. Furthermore, although likely to be rare (with hindsight), it is possible that symptoms attributed to myeloma or lymphoma by interviewees may in fact be unrelated to their cancer diagnosis.

Comparison to other studies

We were unable to identify any other qualitative studies exploring the specific experiences of patients with myeloma and only two targeting lymphoma[37,38]. The latter were both UK-based, and also reported descriptions from patients of challenging pathways and difficulties achieving diagnosis. Other quantitative work reports multiple GP appointments and delay before diagnosis (particularly among myeloma patients); as well as the need to improve time-to-diagnosis[18–23]. Further studies have identified a group of cancers that are 'harder/difficult to suspect'[18,23]. Myeloma is amongst this group due to its propensity for non-specific symptoms commonly found in the general population (e.g. back pain and fatigue[19]). Other 'harder to suspect' malignancies include pancreatic, stomach, lung, colon, brain and ovarian cancers, and Hodgkin lymphoma, and also typically have vague and intermittent symptoms, as well as complex pathways to diagnosis, including multiple GP consultations before hospital referral, more emergency presentations and longer time-to-diagnosis[18,23,39–44]. Conversely malignancies with specific signs and symptoms (e.g. breast cancer and breast lumps; melanoma and skin lesions), or affecting organs that can easily be examined (e.g. testicular or thyroid cancer), generally have fewer GP consultations, more two-week wait referrals for suspected cancer, fewer routine referrals and shorter time-to-diagnosis[18,23,39,40,43]. Given these differences, it is highly likely that the latter group will experience the linear compartmentalisation depicted in the MPT, whereas the former group are more pre-disposed to the chaotic pathways described in this study.

Meaning of the study and implications for practice

Our study clearly depicts the complexity of pathways to lymphoma and myeloma diagnosis. It clarifies the difficulties experienced in trying to identify the cause of what are often non-specific symptoms (intermittent, vague, slowly progressive, common in other illnesses). This occurred often despite the best efforts of patients, relatives and HCPs, including patients re-presenting to their GPs for reassessment, and GPs requesting numerous investigations, which were often normal (including blood tests, imaging and colonoscopy/endoscopy, the latter often initiated due to anaemia). Our findings highlight some of the reasons for delays in help-seeking, re-presentation and diagnosis[18–22], as well as potential explanations for the lack of urgent hospital referrals for suspected cancer and the high proportion of emergency presentations[17,24–26]. Our results also explain why interventions such as education campaigns targeting the (very broad) symptoms of these diseases, would probably lack impact. Interestingly, some patients reported symptoms that were present before and after diagnosis (e.g. nocturia: L24). Whilst such symptoms were considered significant at the time, persistence after successful chemotherapy is likely to indicate the issue was unrelated to their lymphoma, thus highlighting the difficulty of distinguishing malignant from benign disease. Moving forward, and in the absence of clear symptom signatures and blood tests[45–47] (although certain tests can rule myeloma in or out[48]), it is important that the complexity experienced by patients with myeloma and lymphoma, as well as other 'harder to suspect' cancers, is acknowledged within strategies to reduce time-to-diagnosis, including those set out in the NHS Long Term Plan[7]. Importantly, this document introduced Rapid Diagnostic Centres (RDCs) that provides GPs with an

For peer review only - http://bmjopen.bmj.com/site/about/guidelines.xhtml

alternative fast access pathway to secondary care for patients with symptoms that are worrying, but non-specific, so do not suggest referral to a specific speciality. This could represent a significant change in the context of myeloma and lymphoma, as well as other 'harder to suspect' cancers, particularly if used in conjunction with rigorous safety netting in primary care[4,49], of which some evidence was found in the current study. For members of the public, individual recognition of their own 'normal' is key, as well as having the confidence to pursue help-seeking for unexplained symptoms.

Unanswered questions and future research

Primary care HCPs have significant input into the care of patients later diagnosed with cancer, including those with emergency presentation[50]. To our knowledge, however, there have been no qualitative studies exploring GP experiences of managing patients with lymphoma or myeloma. Nor is there any work linking primary and secondary healthcare data, although such studies would clarify the occurrence and timing of events between symptom onset and diagnosis and promote understanding of these complex pathways. Exploring the impact of RDCs on time-to-diagnosis and disease stage at diagnosis will provide insights into the efficacy of this new pathway for patients with myeloma and lymphoma.

Figure 1 Model of Pathways to Treatment[27]

Figure 2: Summary of key themes and components

For Peer review only

Declarations

Funding: This study was funded by Cancer Research UK (National Awareness and Early Diagnosis Initiative: C38860/A13509); Bloodwise (15037); and the National Institute for Health Research Programme Grant for Applied Research: RP-PG-0613-20002.

Ethical approval: REC 04/01205/69 (HMRN); REC 12/YH/0149 (Qualitative study). The study was also registered with the UK Clinical Research Network Portfolio: UKCRN ID:11963.

Competing interests: The authors have declared no competing interests.

Authors contributions: DH, ER, AS, UM and RP devised the study, the methods and secured funding. RH and DH conducted interviews and analysed the data. UM and RP provided clinical input. DH prepared the draft manuscript. All authors edited and approved the final manuscript.

Data sharing: Ethical approvals do not permit data sharing. Patients did not consent to data sharing. The corresponding author can be contacted for further information.

Acknowledgements: We are grateful to the patients and relatives who took part in this study and shared their experiences with us at a difficult time.

For peer review only

For peer review only - http://bmjopen.bmj.com/site/about/guidelines.xhtml

References

1. Neal, R. D. *et al.* Is increased time to diagnosis and treatment in symptomatic cancer associated with poorer outcomes? Systematic review. *Br. J. Cancer* **112 Suppl 1**, S92-107 (2015).
2. Tørring, M. L. *et al.* Evidence of advanced stage colorectal cancer with longer diagnostic intervals: a pooled analysis of seven primary care cohorts comprising 11 720 patients in five countries. *Br. J. Cancer* **117**, 888–897 (2017).
3. NICE. *Referral Guidelines for Suspected Cancer*. (2005).
4. NICE. *Suspected cancer: recognition and referral*. https://www.nice.org.uk/guidance/ng12/resources/suspected-cancer-recognition-and-referral-1837268071621 (2015).
5. Department of Health. *The NHS Cancer Plan: A plan for investment; A plan for reform*. (2000).
6. Neal, R. D. *et al.* Comparison of cancer diagnostic intervals before and after implementation of NICE guidelines: analysis of data from the UK General Practice Research Database. *Br. J. Cancer* **110**, 584–592 (2014).
7. NHS England. *NHS Long Term Plan*. https://www.longtermplan.nhs.uk/publication/nhs-long-term-plan/ (2019).
8. ACE Programme. *Key messages from the evaluation of Multidisciplinary Diagnostic Centres (MDS): A new approach to the diagnosis of cancer*. (2019).
9. Lewis, R. *Improving diagnostic pathways for patients with vague symptoms*. https://www.macmillan.org.uk/_images/ace-vague-symptoms-report-final_tcm9-311011.pdf (2017).
10. National Cancer Intelligence Network. *Cancer Incidence and Mortality By Cancer Network, UK, 2005*. www.ncin.org.uk (2008).
11. Westlake, S. Cancer incidence and mortality in the United Kingdom and constituent countries, 2004-06. *Health Stat Q* 56–62 (2009).

For peer review only

For peer review only - http://bmjopen.bmj.com/site/about/guidelines.xhtml

12. Jemal, A., Siegel, R., Xu, J. & Ward, E. Cancer statistics, 2010. *CA Cancer J Clin* **60**, 277–300 (2010).

13. Jemal, A. *et al.* Global cancer statistics. *CA Cancer J Clin* **61**, 69–90 (2011).

14. Swerdlow, S. *et al. WHO Classification of Tumours of Haematopoietic and Lymphoid Tissues*. (World Health Organization, 2017).

15. Whitaker, K. L. *et al.* The association between normalising attributions and help-seeking for cancer alarm symptoms in the community: a cross-sectional survey. *The Lancet* **384, Supplement 2**, S86 (2014).

16. Howell, D. A. *et al.* Disease-related factors affecting timely lymphoma diagnosis: a qualitative study exploring patient experiences. *Br J Gen Pract* **69**, e134–e145 (2019).

17. Howell, D. A. *et al.* Myeloma: Patient accounts of their pathways to diagnosis. *PLOS ONE* **13**, e0194788 (2018).

18. Lyratzopoulos, G., Abel, G. A., McPhail, S., Neal, R. D. & Rubin, G. P. Measures of promptness of cancer diagnosis in primary care: secondary analysis of national audit data on patients with 18 common and rarer cancers. *British Journal of Cancer* **108**, 686–690 (2013).

19. Howell, D. A. *et al.* Time-to-diagnosis and symptoms of myeloma, lymphomas and leukaemias: a report from the Haematological Malignancy Research Network. *BMC Blood Disorders* **13**, 9 (2013).

20. Keeble, S. *et al.* Variation in promptness of presentation among 10,297 patients subsequently diagnosed with one of 18 cancers: Evidence from a National Audit of Cancer Diagnosis in Primary Care. *Int. J. Cancer* **135**, 1220–1228 (2014).

21. Din, N. U. *et al.* Age and Gender Variations in Cancer Diagnostic Intervals in 15 Cancers: Analysis of Data from the UK Clinical Practice Research Datalink. *PLOS ONE* **10**, e0127717 (2015).

22. Koshiaris, C. *et al.* Quantifying intervals to diagnosis in myeloma: a systematic review and meta-analysis. *BMJ Open* **8**, e019758 (2018).

For peer review only - http://bmjopen.bmj.com/site/about/guidelines.xhtml

For peer review only

23. Lyratzopoulos, G., Neal, R. D., Barbiere, J. M., Rubin, G. P. & Abel, G. A. Variation in number of general practitioner consultations before hospital referral for cancer: findings from the 2010 National Cancer Patient Experience Survey in England. *Lancet Oncol.* **13**, 353–365 (2012).
24. Elliss-Brookes, L. *et al.* Routes to diagnosis for cancer – determining the patient journey using multiple routine data sets. *British Journal of Cancer* **107**, 1220–1226 (2012).
25. Howell, D. *et al.* Multiple myeloma: routes to diagnosis, clinical characteristics and survival – findings from a UK population-based study. *Br J Haematol* **177**, 67–71 (2017).
26. Kane, E. *et al.* Emergency admission and survival from aggressive non-Hodgkin lymphoma: A report from the UK's population-based Haematological Malignancy Research Network. *European Journal of Cancer* **78**, 53–60 (2017).
27. Walter, F., Webster, A., Scott, S. & Emery, J. The Andersen Model of Total Patient Delay: a systematic review of its application in cancer diagnosis. *J Health Serv Res Policy* **17**, 110–118 (2012).
28. Weller, D. *et al.* The Aarhus statement: improving design and reporting of studies on early cancer diagnosis. *Br J Cancer* **106**, 1262–1267 (2012).
29. Tong, A., Sainsbury, P. & Craig, J. Consolidated criteria for reporting qualitative research (COREQ): a 32-item checklist for interviews and focus groups. *International Journal for Quality in Health Care* **19**, 349–357 (2007).
30. Barbour, R. *Introducing Qualitative Research*. (Sage, 2019).
31. Sandelowski, M. Focus on Research Methods: Whatever Happened to Qualitative Description? *Res. Nurs. Health* **23**, 334–340 (2000).
32. Sandelowski, M. What's in a name? Qualitative description revisited. *Res. Nurs. Health* **33**, 77–84 (2010).
33. Smith, A. *et al.* The Haematological Malignancy Research Network (HMRN): a new information strategy for population based epidemiology and health service research. *Br J Haematol* **148**, 739–753 (2010).

For peer review only - http://bmjopen.bmj.com/site/about/guidelines.xhtml

34. Smith, A. *et al.* Cohort Profile: The Haematological Malignancy Research Network (HMRN); a UK population-based patient cohort. *Int J Epidemiol* (2018) doi:10.1093/ije/dyy044.

35. Charmaz, K. *Constructing grounded theory: A practical guide through qualitative analysis*. (Sage, 2006).

36. Lincoln YS & Guba EG. *Naturalistic Inquiry*. (Sage Publications, 1985).

37. Howell, D. A., Smith, A. G. & Roman, E. Help-seeking behaviour in patients with lymphoma. *Eur J Cancer Care (Engl)* **17**, 394–403 (2008).

38. Wall, C., Glenn, S. & Poole, H. Experiences prior to diagnosis of non-Hodgkin lymphoma: a phenomenological study. *J Adv Nurs* **67**, 2363–2372 (2011).

39. Lyratzopoulos, G. *et al.* The relative length of the patient and the primary care interval in patients with 28 common and rarer cancers. *Br. J. Cancer* **112 Suppl 1**, S35-40 (2015).

40. Swann, R. *et al.* Diagnosing cancer in primary care: results from the National Cancer Diagnosis Audit. *Br J Gen Pract* **68**, e63–e72 (2018).

41. Walter, F. M. *et al.* Symptoms and patient factors associated with diagnostic intervals for pancreatic cancer (SYMPTOM pancreatic study): a prospective cohort study. *The Lancet Gastroenterology & Hepatology* **1**, 298–306 (2016).

42. Walter, F. M. *et al.* Symptoms and other factors associated with time to diagnosis and stage of lung cancer: a prospective cohort study. *British Journal of Cancer* **112**, S6–S13 (2015).

43. Koo, M. M., Hamilton, W., Walter, F. M., Rubin, G. P. & Lyratzopoulos, G. Symptom Signatures and Diagnostic Timeliness in Cancer Patients: A Review of Current Evidence. *Neoplasia* **20**, 165–174 (2017).

44. Evans, J., Chapple, A., Salisbury, H., Corrie, P. & Ziebland, S. 'It can't be very important because it comes and goes'--patients' accounts of intermittent symptoms preceding a pancreatic cancer diagnosis: a qualitative study. *BMJ Open* **4**, e004215 (2014).

For peer review only - http://bmjopen.bmj.com/site/about/guidelines.xhtml

For peer review only

45. Shephard, E. A. *et al.* Quantifying the risk of multiple myeloma from symptoms reported in primary care patients: a large case–control study using electronic records. *British Journal of General Practice* **65**, e106–e113 (2015).

46. Shephard, E. A., Neal, R. D., Rose, P. W., Walter, F. M. & Hamilton, W. T. Quantifying the risk of Hodgkin lymphoma in symptomatic primary care patients aged ≥40 years: a case–control study using electronic records. *British Journal of General Practice* **65**, e289–e294 (2015).

47. Shephard, E. A., Neal, R. D., Rose, P. W., Walter, F. M. & Hamilton, W. T. Quantifying the risk of non-Hodgkin lymphoma in symptomatic primary care patients aged ≥40 years: a large case–control study using electronic records. *British Journal of General Practice* **65**, e281–e288 (2015).

48. Koshiaris, C. *et al.* Early detection of multiple myeloma in primary care using blood tests: a case–control study in primary care. *Br J Gen Pract* bjgp18X698357 (2018) doi:10.3399/bjgp18X698357.

49. Safety netting. *Cancer Research UK* https://www.cancerresearchuk.org/health-professional/diagnosis/suspected-cancer-referral-best-practice/safety-netting (2014).

50. Mitchell, E. D., Rubin, G., Merriman, L. & Macleod, U. The role of primary care in cancer diagnosis via emergency presentation: qualitative synthesis of significant event reports. *Br J Cancer* **112**, S50–S56 (2015).

For peer review only

For peer review only - http://bmjopen.bmj.com/site/about/guidelines.xhtml

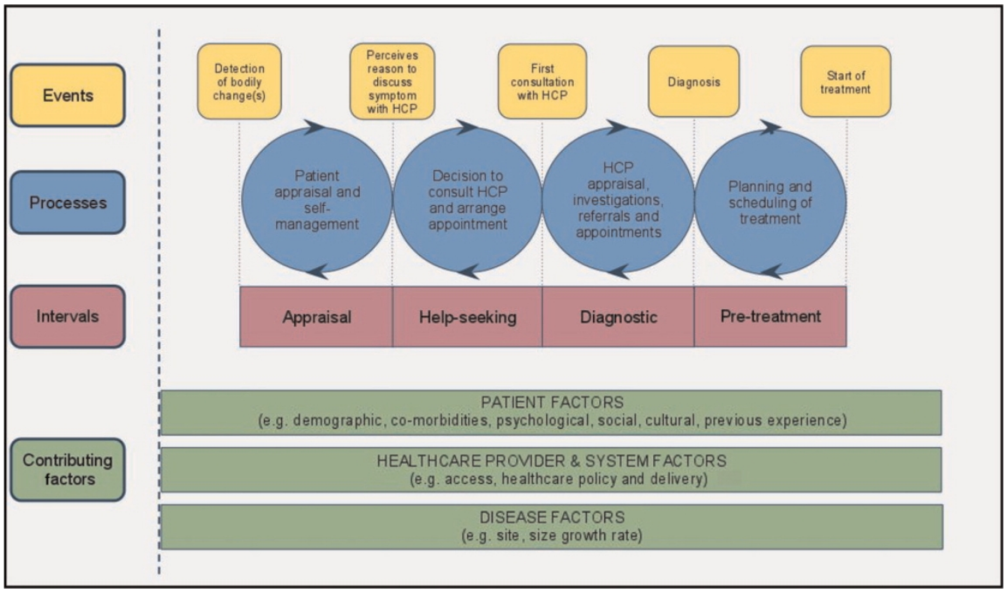

165x96mm (300 x 300 DPI)

Theme 1: Appraisal and re-appraisal

- Ongoing (intermittent or continuous), from symptom onset to diagnosis
- Became more precise and formal over time (use of diaries to document changes etc.)
- Various other explanations for symptoms considered ('normalisation', self-limiting conditions)
- Information sought independently (internet, relatives, friends, colleagues etc.)
- Relatives were involved and adapted their behaviour to the patient's symptoms

Theme 2: Patient initiated self management/treatment

- Ongoing (intermittent or continuous), from symptom onset to diagnosis
- Coping mechanisms (distraction techniques, use of aids such as sticks, wheelchairs)
- Physical adaptation (stopping work, driving, hobbies)
- Lifestyle changes (healthy diet, exercise)
- Paying for treatments (over the counter medication, physiotherapy, osteopath)

Theme 3: Initial help-seeking

- All patients sought help from healthcare professionals (HCPs)
- Impacted by advice from relatives, friends, colleagues etc.
- Broad health checks and general interventions requested
- Could lead to identification of abnormality/suspected cancer and rapid investigation
- Various other explanations for symptoms considered ('normalisation', self-limiting conditions)

Theme 4: Re-presentation

- Common
- Could occur on numerous occasions
- Response to:
    - worsening, progressive, new or alarming symptoms
    - inability to perform 'normal' activities
    - a growing feeling 'something' was wrong, but not knowing exactly what
    - lack of progress towards diagnosis
- Associated with negative investigations (blood tests, radiology, endoscopy, colonoscopy)
- Follow-up could be requested by the HCP (safety netting)
- Dependent on initial advice/diagnosis given to patient by HCP
- Seek advice from various HCPs (dentists, physiotherapists, complementary therapists)
- Emergency route (sent to A&E by HCP, NHS helpline etc.)

Theme 5: Patient initiated actions, decisions and emotions during re-presentation

- Requested more specific help (appraisal of specific sites, particular investigations)
- Evidence presented to show symptom severity/persistence (diary, internet material)
- Giving GPs just the information the patient thought they would consider important
- Attend the same, a different or a specifically identified GP
- Hospital referral/admission requested
- Emotional outbursts
- Involvement of relatives/others to advocate on behalf of the patient
- Use of Emergency route (self-referral to A&E instigated by patient)
- Use of private healthcare to access specialist assessment

For peer review only

Supplementary Table S1: Patient identification, diagnosis, sex, age, time to diagnosis (estimated) and relative's involvement

Patient ID	Diagnosis	Gender	Age[1] (Years)	Time to diagnosis (months)			Relative took part in interview (Y/N)
				T1[2]	T2[3]	T3[4]	
L01[5]	Follicular lymphoma	M	66	1	1.5	2.5	Y
L02	Diffuse large B-cell lymphoma	F	48	1	2	3	N
L03	Follicular lymphoma	F	63	1	3	4	N
L04	Diffuse large B-cell lymphoma	F	64	1.5	1.5	3	N
L05	Marginal zone lymphoma	F	57	0.5	3	3.5	N
L06	Follicular lymphoma	M	39	2	6.5	8.5	N
L07	Classical Hodgkin lymphoma	F	36	0.5	12	12.5	N
L08	Diffuse large B-cell lymphoma	M	60	13	2	15	Y
L09	Diffuse large B-cell lymphoma	F	62	0.5	6	6.5	N
L10	Follicular lymphoma	M	80	0.5	10	10.5	Y
L11	Mantle cell lymphoma	M	71	0.5	10	10.5	Y
L12	Follicular lymphoma	F	84	0.5	1.5	2	R
L13	Follicular lymphoma	M	63	12	3	15	N
L14	Classical Hodgkin lymphoma	M	33	0.5	4	4.5	Y
L15	Mantle cell lymphoma	F	75	6	2	8	Y
L16	Marginal zone lymphoma	M	64	9	5	14	N
L17	Classical Hodgkin lymphoma	F	37	12	1	13	N
L18	Follicular lymphoma	M	53	1	12	13	N
L19	Classical Hodgkin lymphoma	M	56	2	5	7	Y
L20	Diffuse large B-cell lymphoma	M	64	1	5	6	Y
L21	Diffuse large B-cell lymphoma	F	81	0.5	6	6.5	N
L22	Follicular lymphoma	F	52	0.5	2	2.5	N
L23	Classical Hodgkin lymphoma	M	23	0.5	3	3.5	N
L24	Diffuse large B-cell lymphoma	M	75	1	10	11	Y
L25	Marginal zone lymphoma	F	76	0.5	20	20.5	N
L26	Follicular lymphoma	F	63	8	6	14	N
L27	Diffuse large B-cell lymphoma	M	64	1	1	2	Y
L28	Marginal zone lymphoma	F	76	11	3	14	Y
L29	Diffuse large B-cell lymphoma	M	60	0.5	1.5	2	Y
L30	Marginal zone lymphoma	M	60	0.5	25	25.5	N
L31	Diffuse large B-cell lymphoma	M	65	2	15	17	N
L32	Diffuse large B-cell lymphoma	M	64	0.5	3	3.5	Y
L33	Marginal zone lymphoma	F	60	1	14	15	Y
L34	Mantle cell lymphoma	F	70	0.5	2	2.5	N
L35	Diffuse large B-cell lymphoma	F	69	8	1	9	Y
Myeloma 01[6]	Myeloma	M	67	1	2.5	3.5	Y
M02	Myeloma	M	63	2.5	7	9.5	Y
M03	Myeloma	M	71	1	17	18	Y
M04	Myeloma	F	78	1	5	6	N
M05	Myeloma	F	55	1	4	5	N
M06	Myeloma	F	71	3	1.5	2.5	Y
M07	Myeloma	F	56	1	8	11	N
M08	Myeloma	M	68	1	3	4	Y
M09	Myeloma	M	70	1	2	3	N
M10	Myeloma	M	74	2	0.5	2.5	Y
M11	Myeloma	M	59	3	15	18	N
M12	Myeloma	M	43	3	8	11	Y
M13	Myeloma	M	71	1	7	8	N
M14	Myeloma	F	59	1	8	9	Y
M15	Myeloma	M	58	1	17	18	Y
M16	Myeloma	M	66	3	15	18	N
M17	Myeloma	M	70	0.5	2	2.5	N

**M18**	Myeloma	M	71	1	11	12	Y
**M19**	Myeloma	M	62	1	1	2	Y
**M20**	Myeloma	F	74	7	5	12	Y

[1]Age at interview; [2]T1: Initial symptom/health change (estimated from information in the routine self-reported HMRN questionnaire) to first help seeking (appraisal and help-seeking interval); [3]T2: First help-seeking to diagnosis (diagnostic interval): [4]T3: Initial symptom/health change to diagnosis (total interval); [5]Lymphoma patient ID 01; [6]Myeloma patient ID 01.

For peer review only

Consolidated criteria for reporting qualitative studies (COREQ): 32-item checklist

Developed from:
Tong A, Sainsbury P, Craig J. Consolidated criteria for reporting qualitative research (COREQ): a 32-item checklist for interviews and focus groups. *International Journal for Quality in Health Care*. 2007. Volume 19, Number 6: pp. 349 – 357

YOU MUST PROVIDE A RESPONSE FOR ALL ITEMS. ENTER N/A IF NOT APPLICABLE

No. Item	Guide questions/description	Section/Page #
**Domain 1: Research team and reflexivity**		
Personal Characteristics		
1. Interviewer/facilitator	Which author/s conducted the interview or focus group?	DH & RH<br>P6 (Methods)
2. Credentials	What were the researcher's credentials? E.g. PhD, MD	DH: PhD, RGN<br>RH: MA
3. Occupation	What was their occupation at the time of the study?	DH: SRF<br>RH: RF
4. Gender	Was the researcher male or female?	Both female
5. Experience and training	What experience or training did the researcher have?	DH: Registered nurse, Researcher (20 years)<br>RH: Researcher (15 years)
Relationship with participants		
6. Relationship established	Was a relationship established prior to study commencement?	No, P6 (Methods)
7. Participant knowledge of the interviewer	What did the participants know about the researcher? e.g. personal goals, reasons for doing the research	Nothing, P6 (Methods)
8. Interviewer characteristics	What characteristics were reported about the interviewer/facilitator? e.g. Bias, assumptions, reasons and interests in the research topic	None
**Domain 2: study design**		
Theoretical framework		
9. Methodological orientation and Theory	What methodological orientation was stated to underpin the study? e.g. grounded theory, discourse analysis, ethnography, phenomenology, content analysis	P4 (Methods)
Participant selection		
10. Sampling	How were participants selected? e.g. purposive, convenience, consecutive, snowball	P4 (Methods)

For peer review only

11. Method of approach	How were participants approached? e.g. face-to-face, telephone, mail, email	P4 (Methods)
12. Sample size	How many participants were in the study?	P4 (Methods)
13. Non-participation	How many people refused to participate or dropped out? Reasons?	P4 (Methods)
Setting		
14. Setting of data collection	Where was the data collected? e.g. home, clinic, workplace	P6 (Methods)
15. Presence of non-participants	Was anyone else present besides the participants and researchers?	Relatives, who also participated, P4 (Methods), Table S1
16. Description of sample	What are the important characteristics of the sample? e.g. demographic data, date	Table 1, P5 Table S1
Data collection		
17. Interview guide	Were questions, prompts, guides provided by the authors? Was it pilot tested?	Table 2, P6
18. Repeat interviews	Were repeat interviews carried out? If yes, how many?	N/A
19. Audio/visual recording	Did the research use audio or visual recording to collect the data?	Yes, P6 (Methods)
20. Field notes	Were field notes made during and/or after the interview or focus group?	Yes, P6 (Methods)
21. Duration	What was the duration of the interviews or focus group?	P6 (Methods)
22. Data saturation	Was data saturation discussed?	Yes, P4 (Methods) P12 (Discussion)
23. Transcripts returned	Were transcripts returned to participants for comment and/or correction?	No
**Domain 3: analysis and findings**		
Data analysis		
24. Number of data coders	How many data coders coded the data?	P6 (Methods)
25. Description of the coding tree	Did authors provide a description of the coding tree?	No, but this is summarized in Figure 2
26. Derivation of themes	Were themes identified in advance or derived from the data?	P6 (Methods)
27. Software	What software, if applicable, was used to manage the data?	N/A
28. Participant checking	Did participants provide feedback on the findings?	Corroborated by others with similar experiences at patient support group.
Reporting		
29. Quotations presented	Were participant quotations presented to illustrate the themes/findings? Was each quotation identified? e.g. participant number	Yes, P6-12 (Results)

For peer review only

30. Data and findings consistent	Was there consistency between the data presented and the findings?	Yes, P12-14 (Discussion)
31. Clarity of major themes	Were major themes clearly presented in the findings?	Yes, P6-12 (Results)
32. Clarity of minor themes	Is there a description of diverse cases or discussion of minor themes?	Yes, P12-14, (Discussion)

Once you have completed this checklist, please save a copy and upload it as part of your submission. When requested to do so as part of the upload process, please select the file type: *Checklist*. You will NOT be able to proceed with submission unless the checklist has been uploaded. Please DO NOT include this checklist as part of the main manuscript document. It must be uploaded as a separate file.

For peer review only

Standards for Reporting Qualitative Research (SRQR)*

http://www.equator-network.org/reporting-guidelines/srqr/

Page/line no(s).

Title and abstract

**Title** - Concise description of the nature and topic of the study Identifying the study as qualitative or indicating the approach (e.g., ethnography, grounded theory) or data collection methods (e.g., interview, focus group) is recommended	P1
**Abstract** - Summary of key elements of the study using the abstract format of the intended publication; typically includes background, purpose, methods, results, and conclusions	P1

Introduction

**Problem formulation** - Description and significance of the problem/phenomenon studied; review of relevant theory and empirical work; problem statement	P3
**Purpose or research question** - Purpose of the study and specific objectives or questions	P3

Methods

**Qualitative approach and research paradigm** - Qualitative approach (e.g., ethnography, grounded theory, case study, phenomenology, narrative research) and guiding theory if appropriate; identifying the research paradigm (e.g., postpositivist, constructivist/ interpretivist) is also recommended; rationale**	P4
**Researcher characteristics and reflexivity** - Researchers' characteristics that may influence the research, including personal attributes, qualifications/experience, relationship with participants, assumptions, and/or presuppositions; potential or actual interaction between researchers' characteristics and the research questions, approach, methods, results, and/or transferability	P6
**Context** - Setting/site and salient contextual factors; rationale**	P4
**Sampling strategy** - How and why research participants, documents, or events were selected; criteria for deciding when no further sampling was necessary (e.g., sampling saturation); rationale**	P4
**Ethical issues pertaining to human subjects** - Documentation of approval by an appropriate ethics review board and participant consent, or explanation for lack thereof; other confidentiality and data security issues	P15
**Data collection methods** - Types of data collected; details of data collection procedures including (as appropriate) start and stop dates of data collection and analysis, iterative process, triangulation of sources/methods, and modification of procedures in response to evolving study findings; rationale**	P6

For peer review only - http://bmjopen.bmj.com/site/about/guidelines.xhtml

**Data collection instruments and technologies** - Description of instruments (e.g., interview guides, questionnaires) and devices (e.g., audio recorders) used for data collection; if/how the instrument(s) changed over the course of the study	P6, Table 2
**Units of study** - Number and relevant characteristics of participants, documents, or events included in the study; level of participation (could be reported in results)	P5, Table 1 Table S1
**Data processing** - Methods for processing data prior to and during analysis, including transcription, data entry, data management and security, verification of data integrity, data coding, and anonymization/de-identification of excerpts	P6
**Data analysis** - Process by which inferences, themes, etc., were identified and developed, including the researchers involved in data analysis; usually references a specific paradigm or approach; rationale**	P6
**Techniques to enhance trustworthiness** - Techniques to enhance trustworthiness and credibility of data analysis (e.g., member checking, audit trail, triangulation); rationale**	P6

Results/findings

**Synthesis and interpretation** - Main findings (e.g., interpretations, inferences, and themes); might include development of a theory or model, or integration with prior research or theory	P6-12
**Links to empirical data** - Evidence (e.g., quotes, field notes, text excerpts, photographs) to substantiate analytic findings	P6-12

Discussion

**Integration with prior work, implications, transferability, and contribution(s) to the field -** Short summary of main findings; explanation of how findings and conclusions connect to, support, elaborate on, or challenge conclusions of earlier scholarship; discussion of scope of application/generalizability; identification of unique contribution(s) to scholarship in a discipline or field	P12-14
**Limitations** - Trustworthiness and limitations of findings	P12, Strengths and weaknesses

Other

**Conflicts of interest** - Potential sources of influence or perceived influence on study conduct and conclusions; how these were managed	P15
**Funding** - Sources of funding and other support; role of funders in data collection, interpretation, and reporting	P15

*The authors created the SRQR by searching the literature to identify guidelines, reporting standards, and critical appraisal criteria for qualitative research; reviewing the reference lists of retrieved sources; and contacting experts to gain feedback. The SRQR aims to improve the transparency of all aspects of qualitative research by providing clear standards for reporting qualitative research.

For peer review only

For peer review only - http://bmjopen.bmj.com/site/about/guidelines.xhtml

**The rationale should briefly discuss the justification for choosing that theory, approach, method, or technique rather than other options available, the assumptions and limitations implicit in those choices, and how those choices influence study conclusions and transferability. As appropriate, the rationale for several items might be discussed together.

Reference:

O'Brien BC, Harris IB, Beckman TJ, Reed DA, Cook DA. **Standards for reporting qualitative research: a synthesis of recommendations.** *Academic Medicine*, Vol. 89, No. 9 / Sept 2014
DOI: 10.1097/ACM.0000000000000388

For peer review only

For peer review only - http://bmjopen.bmj.com/site/about/guidelines.xhtml
